# Polymer Layered Silicate Nanocomposites: A Review

**DOI:** 10.3390/ma2030992

**Published:** 2009-08-20

**Authors:** Vikas Mittal

**Affiliations:** Institute of Chemical and Bioengineering, Department of Chemistry and Applied Biosciences, ETHZ Zurich, 8093, Zurich, Switzerland

**Keywords:** nanocomposites, layered silicates, exfoliation, intercalation, melt intercalation, in-situ intercalative polymerization, thermal stability, gas barrier, mechanical performance, flame retardancy, crystallinity

## Abstract

This review aims to present recent advances in the synthesis and structure characterization as well as the properties of polymer layered silicate nanocomposites. The advent of polymer layered silicate nanocomposites has revolutionized research into polymer composite materials. Nanocomposites are organic-inorganic hybrid materials in which at least one dimension of the filler is less than 100 nm. A number of synthesis routes have been developed in the recent years to prepare these materials, which include intercalation of polymers or pre-polymers from solution, *in-situ* polymerization, melt intercalation etc. The nanocomposites where the filler platelets can be dispersed in the polymer at the nanometer scale owing to the specific filler surface modifications, exhibit significant improvement in the composite properties, which include enhanced mechanical strength, gas barrier, thermal stability, flame retardancy etc. Only a small amount of filler is generally required for the enhancement in the properties, which helps the composite materials retain transparency and low density.

## 1. Introduction 

Polymers are traditionally reinforced with inorganic fillers to improve their properties as well as to reduce costs. These conventional fillers include talc, calcium carbonate, fibers, etc. The achievement of a significant improvement in the composite properties often requires incorporation of a large amount of the filler in the polymer materials, which consequently leads to the loss of transparency of the composites, as well as increase in the bulkiness of the composite materials. Such particulate filled polymers are often classified as microcomposites, based on the dimensions of the phases involved. However, to meet the rising demands of applications of the various materials, functional hybrids of inorganic fillers and polymeric materials are being continuously developed so as to better combine their constituent’s beneficial properties or to induce new ones [1,2]. Polymer nanocomposites are the new class of hybrid materials in this category. Polymer nanocomposites are nanoscale materials, in which at least one of the components has a dimension smaller than 100 nm. They offer an opportunity to explore new behaviors and functionalities beyond those of conventional materials. Nanoparticles often strongly influence the properties of the composites at very low volume fractions. This is mainly due to their small interparticle distances and the conversion of a large fraction of the polymer matrix near their surfaces into an interphase of different properties as well as to the consequent change in morphology [3]. As a result, the desired properties are usually reached at low filler volume fraction, which allows the nanocomposites to retain the macroscopic homogeneity and low density of the polymer. Besides, the geometrical shape of the particles plays an important role in determining the properties of the composites. The nanocomposites can contain inorganic fillers falling into three different categories by the virtue of their primary particle dimensions [4]. When all the three dimensions of the particles are in the nanometer scale, the inorganic fillers have the form of spherical particles like silica particles [5,6]. Fillers with two dimensions in the nanometer scale whereas the third one is in the range of micrometers include carbon nanotubes or whiskers [7,8]. When the filler has two finite dimensions in the range of micrometers, whereas the third dimension is in nanometer scale, the fillers include layered silicate (or aluminosilicate) materials [9]. Though nanocomposites with all the inorganic materials in the three categories have been synthesized and commercially applied to some extent, it is specially the layered silicate based nanocomposites which have attained maximum research attention owing to many advantages of using layered silicate materials as fillers.

Though layered silicate materials are easily available and have also low cost, however, their nanoscale incorporation in the polymer matrices is not straightforward. In most of the instances, surface modification of these materials is required to obtain organically modified silicates or clay which then is more compatible with the organic polymer matrices. The incorporation of the layered silicate materials into the polymer materials has been already known for half a century. In 1950, Carter *et al.* used the organically modified layered silicates for the reinforcement of elastomers [10]. Greenland also showed the incorporation of clays into polyvinyl alcohol in the aqueous solution [11]. Similarly, a number of other studies confirming the potential of the layered silicates in the composites technology were reported. However, it was the work of Toyota researchers for the development of polymeric nanocomposites in early nineties [12,13], in which electrostatically held 1 nm thick layers of the layered aluminosilicates were dispersed in the polyamide matrix on a nanometer level, which led to an exponential growth in the research in these layered-silicate nanocomposites. The route suggested by Toyota researchers was *in-situ* generation of polymer nanocomposites by using monomers. Subsequently, Giannelis and co-workers [14,15] also reported the route of melt intercalation for the synthesis of polymer nanocomposites. In this route of nanocomposite synthesis, high molecular weight polymers were melted at high temperature and the filler was added to the melt. This route became the most preferred way for the generation of nanocomposites, especially with commercially important polymers like polyolefins. Since then, nanocomposites with practically all the polymer matrices have been reported and a number of different manufacturing routes for the delamination of the filler in the polymer matrices have been developed. Substantial improvements in properties like strength, modulus, thermal stability, flame retardency and decrease in gas permeability at very low filler contents as compared to the conventional composites [16,17,18,19,20,21,22,23,24] have been reported. In the aluminosilicate family montmorillonite, which can be easily exfoliated to 1 nm thick platelets in water, has been frequently used to prepare polymer nanocomposites. Though the generation of nanocomposites with nanoscale dispersion of filler in various polymer matrices has been achieved with varying degrees of success, the commercial applications of these nanocomposites are still in infancy and some of the fundamental questions facing nanocomposite technology still need to be answered in order to better control the properties and behavior of these nanocomposites.

## 2. Layered Silicates

**Figure 1:**
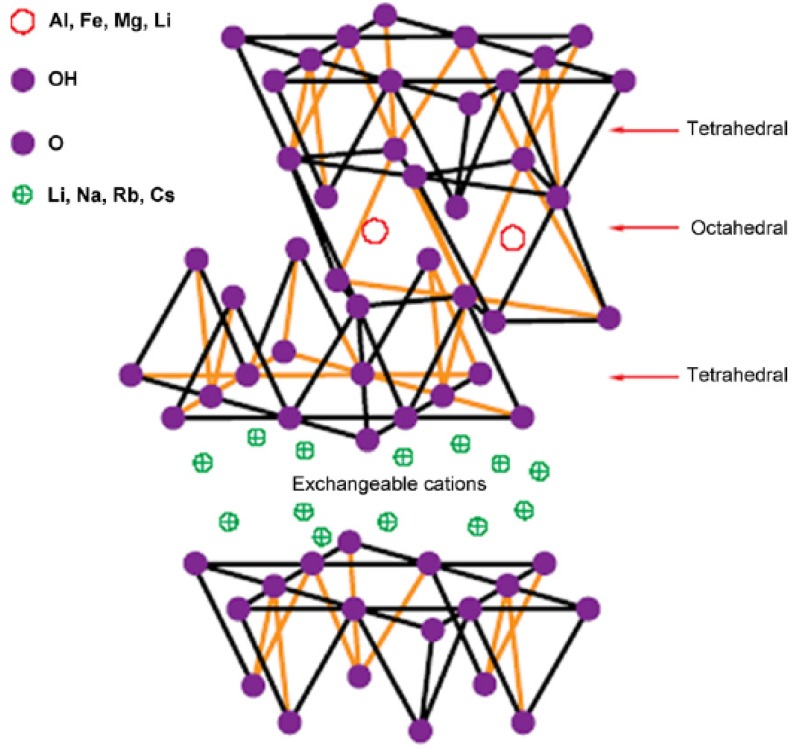
Structure of the 2:1 layered silicates. Reproduced from references [4] and [27] with permission from Elsevier.

As mentioned above, montmorillonite has been a filler of choice for most of the studies on polymer nanocomposites. Montmorillonite is an expandable dioctahedral smectite belonging to the family of the 2:1 phyllosilicates [25,26]. The general formula of montmorillonites is M_x_(Al_4-x_Mg_x_)Si_8_O_20_(OH)_4_. Its particles consist of stacks of 1 nm thick aluminosilicate layers (or platelets) with a regular gap in between (interlayer). Each layer consists of a central Al-octahedral sheet fused to two tetrahedral silicon sheets. In the tetrahedral sheets, silicon is surrounded by four oxygen atoms, whereas in the octahedral sheets, an aluminium atom is surrounded by eight oxygen atoms. Isomorphic substitutions of aluminum by magnesium in the octahedral sheet generate negative charges, which are compensated for by alkaline-earth- or hydrated alkali-metal cations, as shown in Figure 1 [4,27]. The majority of these cations are present in the interlayers between the sheets, but some percentage of them are present on the edges of the sheets. Based on the extent of the substitutions in the silicate crystals, a term called layer charge density is defined. Montmorillonites have a mean layer charge density of 0.25-0.5 equiv.mol^-1^. The layer charge is also not constant and can vary from layer to layer, therefore, it should be considered more of an average value. The electrostatic and van der Waals forces holding the layers together are relatively weak in smectites and the interlayer distance varies depending on the radius of the cation present and its degree of hydration. As a result, the stacks swell in water and the 1 nm thick layers can be easily exfoliated by shearing, giving platelets with high aspect ratio. Unfortunately, their high energetic hydrophilic surfaces are incompatible with many polymers, whose low energetic surfaces are hydrophobic. However, their inorganic cations can be easily exchanged with organic ions (e.g., alkylammonium) to give organically modified montmorillonite (OMMT) that does not suffer from this problem [28,29]. An exchange of inorganic cations with organic cations renders the silicate organophilic and hydrophobic and lowers the surface energy of the platelets and increases the basal-plane or interlayer spacing (d-spacing) [30]. This improves the wetting, swelling, and exfoliation of the aluminosilicate in the polymer matrix. This technology has been also been widely reported by Pinnavaia [31], Krishnamoorti & Giannelis [32], Theng [28] and Lagaly [29]. Alkyl ammonium ions like octadecyltrimethylammonium, dioctadecyldimethylammonium etc. have been conventionally used for the organic modification of silicates.

Owing to the low charge density (0.25-0.5 equiv.mol^-1^) of montmorillonites, a larger area per cation is available on the surface, which leads to a lower interlayer spacing in the modified montmorillonite after surface ion exchange with alkyl ammonium ions. On the other hand, the minerals with high charge density (1 equiv.mol^-1^) like mica have much smaller area per cation and can lead to much higher basal plane spacing after surface modification, however, owing to very strong electrostatic forces present in the interlayers due to the increased number of ions, these minerals do not swell in water and thus do not allow the cation exchange. However, aluminosilicates with medium charge densities of 0.5-0.8 equiv.mol^-1^, like vermiculite, offer a potential of partial swelling in water and cation exchange which can lead to much higher basal plane spacing in the modified mineral if optimum ion exchange is achieved. In the pristine state, vermiculite particles are composed of stacks of negatively charged 2:1 aluminosilicate layers (ca. 0.95 nm thick) with one octahedral sheet sandwiched between two opposing tetrahedral sheets and a resulting regular gap in between (interlayer). The chemical constitution of its unit cell is (Mg,Al,Fe)_3_(Al,Si)_4_O_10_(OH)_2_Mg_x_(H_2_O)_n_ [26,29,33,34]. Due to isomorphic substitutions in the lattice, the layers have permanent negative charges that are compensated mainly by hydrated Mg^2+^ as interlayer cations. Owing to the higher basal plane spacing in the modified mineral, the electrostatic interactions holding the layers together can be expected to be lower than similar montmorillonite counterparts thus increasing the potential of better properties of the hybrid nanocomposites. 

In order to know about the amount of organic ammonium ions required to exchange the inorganic cations present in the silicate interlayers, it is important to measure first the cation exchange capacity (CEC) of the minerals. Although many methods have been developed to ascertain CEC [35,36], a more advanced method has been recently reported which takes into account the exchange rate and reaction kinetics [37,38]. Cu(trien)^2+^ complex was found to have a strong affinity to the aluminosilicates and its concentration could be determined photometrically thus providing a reliable method to determine the cation exchange capacity. These values differ from one mineral to the other corresponding to the different layer charge density of the minerals. 

**Figure 2:**
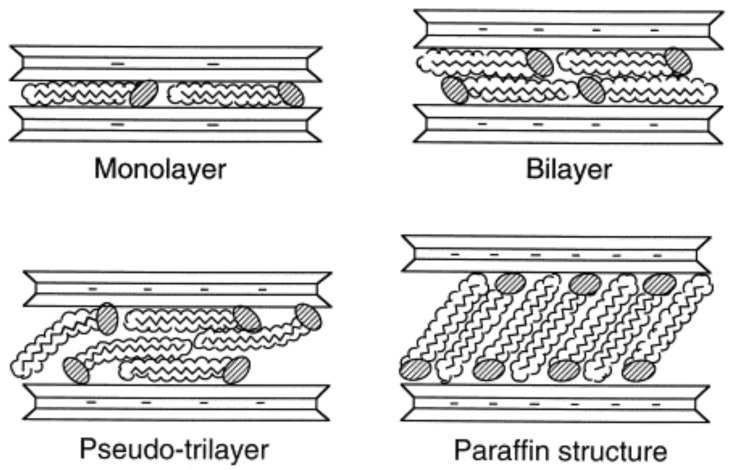
Possible orientations of the alkyl ammonium chains in the interlayers of aluminosilicates as deduced from the X-ray diffraction studies. Reproduced from reference [40] with permission from Elsevier.

In order to describe the structure of the interlayer in organoclays, it is believed that to counter the negative charge on the surface, the cationic head group of the alkylammonium molecule preferentially resides at the layer surface, leaving the organic tail radiating away from the surface. Two parameters then define the equilibrium layer spacing in a given temperature range: the cation exchange capacity of the layered silicate driving the packing of the chains and the chain length of organic tails [12]. According to X-ray diffraction (XRD) data, the organic chains have been long thought to lie either parallel to the silicate layer, forming mono or bilayers or, depending on the packing density and the chain length, to radiate away from the surface, forming pseudo trilayers or even tilted ‘paraffinic’ arrangement [40] as shown in Figure 2. 

**Figure 3:**
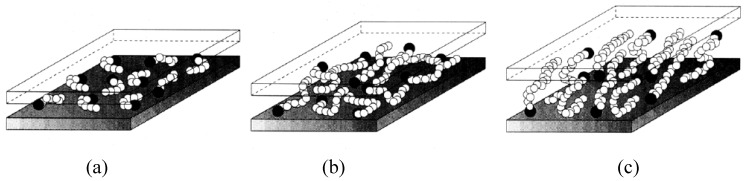
Alkyl chain aggregation models: For the shortest lengths (a), the molecules are effectively isolated from each other. At intermediate lengths (b), quasidiscrete layers form with various degrees of in plane disorder and interdigitation between the layers. For longer lengths (c), interlayer order increases. Reproduced from reference [41] with permission from The American Chemical Society.

**Figure 4:**
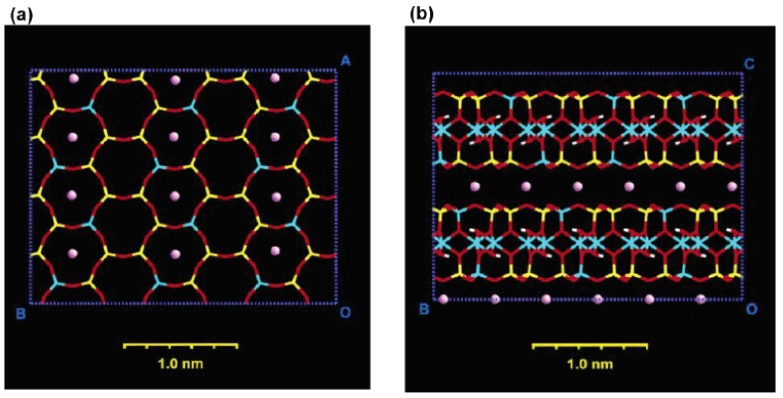
A simulated representation of mica unit cells. Al atoms are depicted in blue, Si in yellow, O in red, H in white, and K (or Li) ions in violet. (a) Top view showing only the upper tetrahedral layer and (b) side view showing that the cavities in the lamellae are fully occupied with alkali ions and stacked on each other. Reproduced from reference [42] with permission from American Chemical Society.

**Figure 5:**
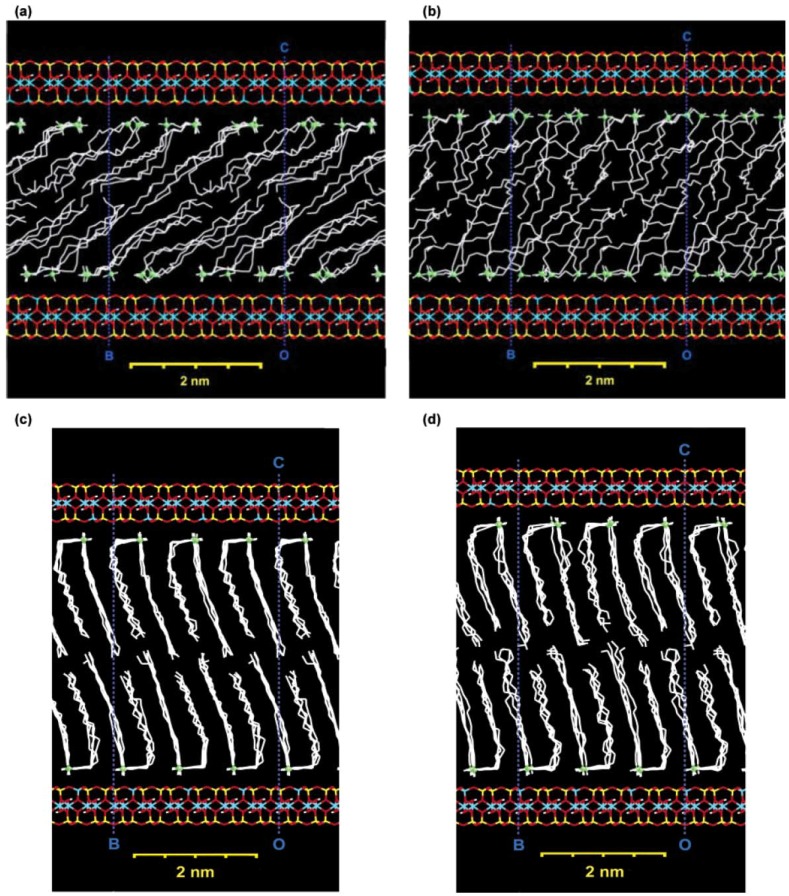
Snapshots of octadecyltrimethylammonium (C18) modified mica and dioctadecyldimethylammonium (2C18) modified mica in MD after 400 ps. (a) C18-mica, 20 °C; (b) C18-mica, 100 °C; (c) 2C18-mica, 20 °C; (d) 2C18-mica, 100 °C. A major conformational change (corresponding to two phase transitions) in C18-layered mica can be seen, whereas in 2C18-mica, only some more gauche-conformations are present at 100 °C and no order-disorder transition occurs due to close packing. Reproduced from reference [42] with permission from The American Chemical Society.

A more realistic description has been proposed by Vaia *et al.* [41], based on FTIR experiments. By monitoring frequency shifts of the asymmetric CH_2_ stretching and bending vibrations, they found that the intercalated chains exist in states with varying degrees of order. In general, as the interlayer packing density or the chain length decreases (or the temperature increases), the intercalated chains adopt a more disordered, liquid like structure resulting from an increase in the gauche/trans conformer ratio. When the available surface area per molecule is within a certain range, the chains are not completely disordered but retain some orientational order similar to that in the liquid crystalline state as shown in Figure 3. Molecular dynamics simulations have also been used to gain further insights into the structures of the layered silicate as well as positioning and behavior of ammonium ions after cation exchange and as a function of temperature. Figure 4 and Figure 5 depict these more realistic simulated models of mica layers before and after alkylammonium surface exchange [42]. The exchange was carried out using octadecyltrimethylammonium and dioctadecyldimethylammonium ions. The effect of temperature on the dynamics of the modified silicates could also be accurately followed. 

**Figure 6:**
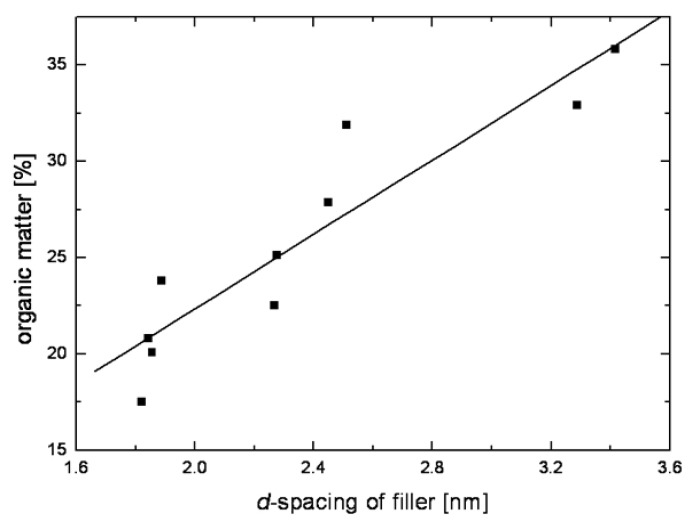
Correlation between the organic matter ionically bound to the layered silicate surface and the resulting basal plane spacing of the modified filler. Reproduced from reference [43].

High resolution thermogravimetric analysis is used to ascertain the amount of organic matter ionically bound to the surface of the layered silicates. The amount of the organic matter in the interlayers can also be correlated to the resulting basal plane spacing in the modified fillers [43] as shown in Figure 6. This correlation thus can be used to tune the surface modifications in order to achieve a certain separation between the filler interlayers.

## 3. Nanocomposite Structures and Characterization

**Figure 7:**
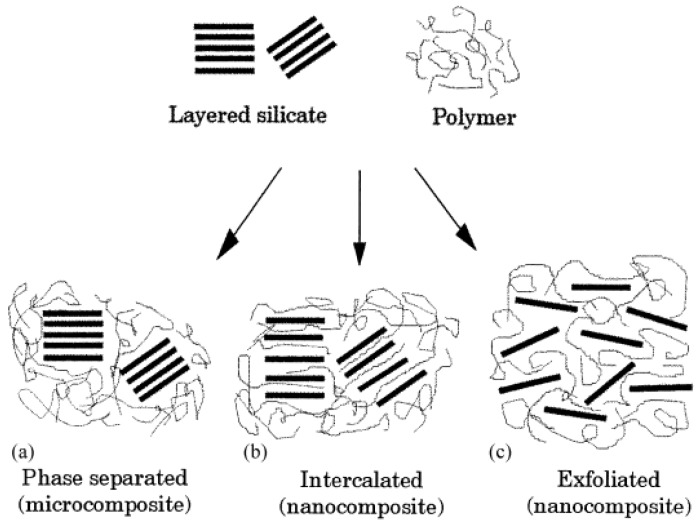
Schematic representation of the different types of composites generated based on the interactions of the layered silicate with polymer matrices. (a) Phase separated microcomposite, (b) intercalated nanocomposite and (c) exfoliated nanocomposite [39].

The microstructure of the composites generated owing to the interactions between the organic polymer and inorganic filler phases is ideally classified as unintercalated (phase separated), intercalated and exfoliated composites, as shown in Figure 7 [39]. The composite microstructure is classified as exfoliated when the filler platelets are completely delaminated into their primary nanometer scale size and the platelets are far apart from each other so that the periodicity of this platelet arrangement is totally lost. This happens when the electrostatic forces of interaction between the platelets have completely been overcome by the polymer chains in the composites. 

When a single or sometimes more than one extended polymer chain is intercalated into the clay interlayers, but the periodicity of the clay platelets is still intact, such a microstructure is termed as intercalated. This structure indicates that though the organic-inorganic hybrid is formed, the electrostatic forces of interaction between the clay platelets could not however be totally dissolved. Based on the interfacial interactions and mode of mixing of the organic and inorganic phases, it is possible that both the phases do not intermix at all and a microcomposite or unintercalated composite is formed. This kind of structure is not at all a nanocomposite and like conventional composites, requires a large amount of filler to achieve significant improvement n the composite properties, which otherwise can be achieved at much lower filler amounts in the case of nanocomposites.

**Figure 8:**
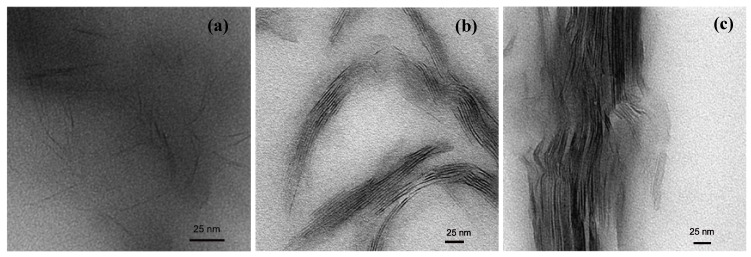
TEM micrographs indicating various possible morphologies in the composites as function of the filler distribution: (a) exfoliated, (b) intercalated and (c) unintercalated. Reproduced from reference [44].

Transmission electron microscopy (TEM) is the most commonly used method to characterize the microstructure of the nanocomposites. Figure 8 shows the TEM micrographs depicting the various idealized morphologies of the polymer nanocomposite structures [44]. Figure 8a represents the exfoliated morphology, where the black lines are the cross-section of the aluminosilicate platelets. The platelets can be seen as single and uniformly dispersed, though they are completely misaligned. On many occasions, the bending and folding of the platelets has also been observed. Figure 8b shows the micrograph with intercalated platelets. The microstructure represents a well ordered multi layer morphology consisting of alternate polymer and inorganic layers. Such a periodicity gives a signal in the X-ray diffractograms and thus the amount of intercalation can then be quantified by the comparison with the basal plane spacing of the modified filler. The exfoliated morphology does not generate any diffraction signal owing to the loss of periodicity and the absence of diffraction peak is taken as the proof of the generation of completely exfoliated nanocomposites. Figure 8c shows the microcomposite or phase separated morphology. Here the filler is not at all intercalated by the polymer chains and the thick filler tactoids are present separated from the polymer phase. However, it should be noticed that these classifications of the composite microstructure as exfoliated and intercalated are not very realistic as in reality, generally a mixture of different morphologies is present. Different extents of intercalation as well exfoliation are generally observed and at maximum, only qualitative classification of the morphology as more or less intercalated or exfoliated can be made. 

**Figure 9:**
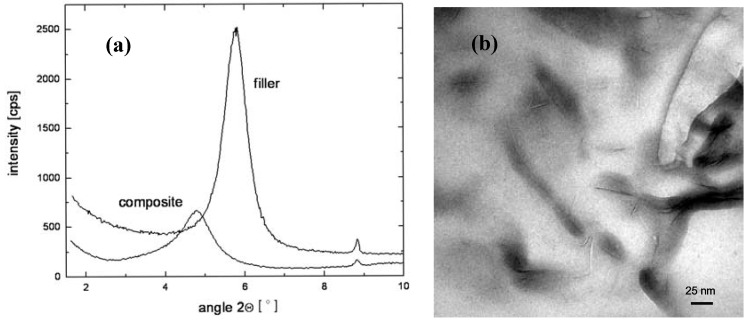
(a) X-ray diffractograms of benzyldibutyl(2-hydroxyethyl)ammonium modified montmorillonite and its nanocomposites with epoxy polymer containing 3 vol% filler and (b) TEM micrograph of the epoxy nanocomposite. Reproduced from reference [44].

Owing to its accuracy and ease of operation, X-ray diffraction (XRD) is one of the most important techniques used for the characterization of the microstructure of the nanocomposites [46]. The XRD is used to quantify the amount of increase in the basal plane spacing in the filler after surface modification as well as after the composite generation. The increased basal plane spacing after the surface exchange leads to the information regarding the possible alignment of the modification molecules inside the clay interlayers, whereas the presence or absence of diffraction peaks in the composites is used to assess information about the microstructure of the composites. Though the XRD also provides the information about the amount of organic matter present in the clay interlayers, however it cannot provide information on the excess of the surface modification molecules present on the clay surface as is provided by thermogravimetric analysis. As mentioned above, the intensity of the X-ray diffractograms is generally taken as a measure to classify the microstructure as intercalated or exfoliated. However, it should be noticed that the X-ray signal are very qualitative in nature and are strongly influenced by the sample preparation, orientation of the platelets as well as defects present in the crystal structure of the montmorillonites. Therefore, the classification of the nanocomposite microstructure just based on the intensity can be faulty. Also, the presence of diffraction signal in the diffractograms of the composite does not mean that 100% of the microstructure is intercalated and it is quite possible to have significant amount of exfoliation present in the composite. Similarly, absence of diffraction signal also does not guarantee the complete exfoliation as small or randomly oriented intercalated platelets may still be present in the composite. As an example, in Figure 9 are shown the X-ray diffractograms of the montmorillonite modified with benzyldibutyl(2- hydroxyethyl)ammonium and its composite with epoxy [44]. The increase in the basal plane spacing of the filler after the composite synthesis was minimal and the diffraction signal of the filler in composite material was quite strong in the diffractograms indicating the intercalated structure. However, when investigated with transmission electron microscopy, a large part of the filler was exfoliated and single platelets were uniformly distributed in the polymer matrix [45]. Therefore, it should be noted that the X-ray diffraction signal are qualitative in nature and idealized classifications of the composite morphologies into interacted or exfoliated is very arbitrary.

**Figure 10:**
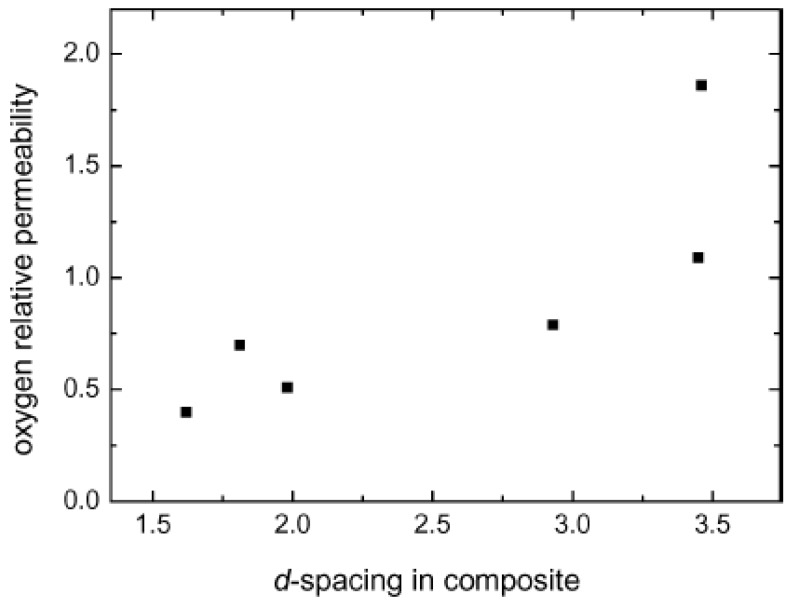
Correlation of the relative oxygen permeability through the nanocomposite films as a function of basal plane spacing of the filler in the composites. Reproduced from reference [45] with permission from The American Chemical Society.

**Figure 11:**
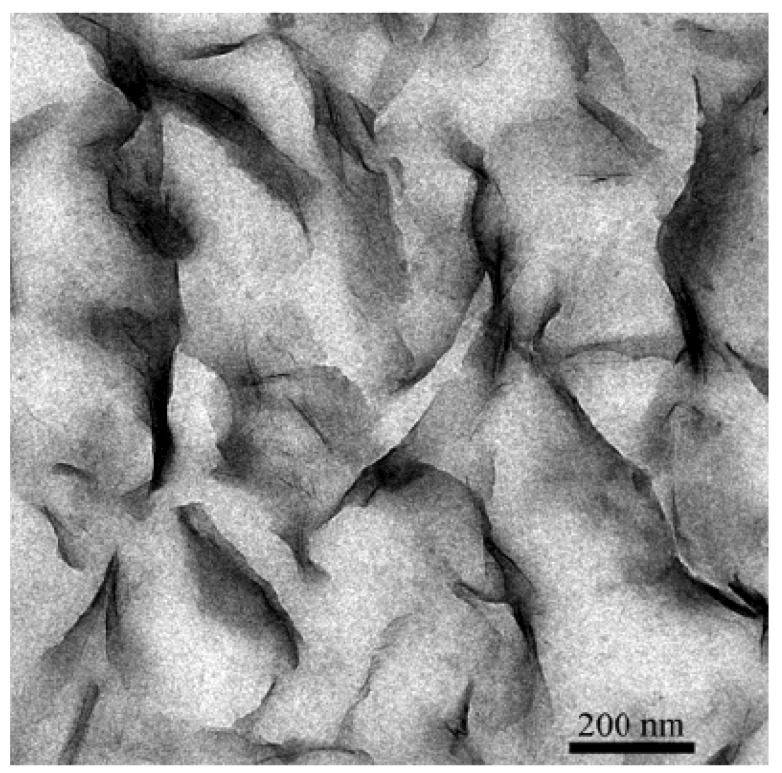
TEM micrograph of the epoxy composite containing 3 vol% of the filler. Reproduced from reference [45] with permission from The American Chemical Society.

It is also to be noted that the achievement of exfoliation leads to the maximum improvement of the properties as it is increase in the aspect ratio of the filler particles after exfoliation which leads to the improvement potential in the composite material. The intercalated platelets though can enhance the properties to some extent but the aspect ratio does not increase owing to the presence of periodicity of platelets, therefore, the interfacial interactions between the organic and filler phases are still not optimum [45]. To confirm this point, increase in the basal plane spacing of the filler in the epoxy composites was plotted as a function of the oxygen permeation through the composite films containing the layered silicates modified with different surface modifications as shown in Figure 10. The oxygen permeation is apparently observed to increase by the increase in the basal plane spacing of the filler which is exactly opposite to the expected behavior as increase in the basal plane spacing should lead to better improvement in the properties and in this case reduction in the oxygen permeation. It is thus clear that it is not the basal plane spacing of the filler or its increase by the intercalation of the filler, but the exfoliated layered silicate platelets which impact the composite properties. Thus, the final aspect ratio of the filler platelets is the important parameter which should be aimed to be increased as much as possible. However, the evaluation of the aspect ratio of the filler platelets in the composite material is also not straightforward owing to the bending and folding of the platelets as shown in Figure 11. In many reported studies, the TEM micrographs have been used to generate information on the average aspect ratio, but in the light of complexities associated with the evaluation of the aspect ratio from the micrographs, other means to obtain an average aspect ratio of the filler in the composites are required.

**Figure 12:**
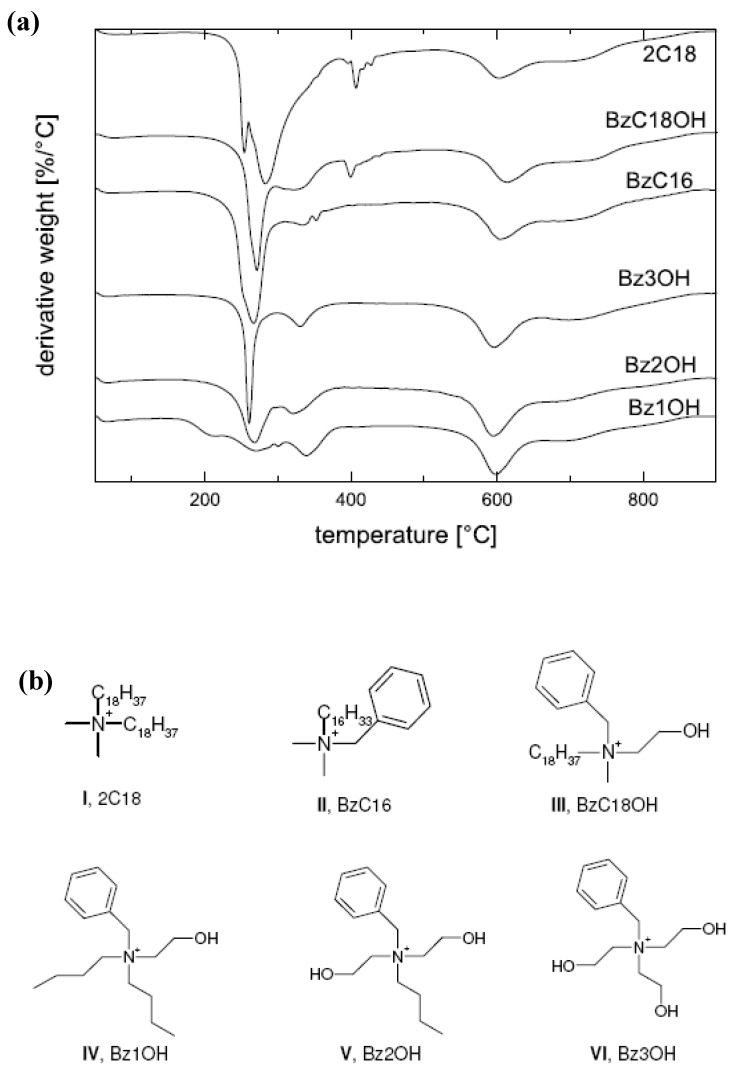
(a) TGA thermograms of the modified fillers with various ammonium ions and (b) chemical structures of the ammonium ions exchanged on the filler surface. Reproduced from reference [45] with permission from The American Chemical Society.

High-resolution (Hi-Res) thermogravimetric analysis (TGA) of the modified fillers and nanocomposites, in which the heating rate is coupled to the mass loss, that is, the sample temperature is not raised until the mass loss at a particular temperature is completed, is another means of characterization of the modified fillers as well as synthesized composites. For the modified fillers, it helps to quantify the amount of the organic matter present in the filler interlayers. Quantitative analysis of the organic monolayer on the montmorillonite surface is achieved by subtracting the mass loss due to high temperature dehydroxylation of the mineral from the total mass loss. In addition, the weight loss between 50 °C and 150 °C corresponding to the evaporation of physisorbed water and solvent molecules is also subtracted from the total weight loss. The total exchanged moles of the ammonium cation per gram of clay *φamm*, is then calculated using the following expression:

φamm = Wcorr/[(1- Wcorr) * Mamm]

where *Wcorr* is the corrected mass loss corresponding to organic weight loss owing to only alkyl ammonium ions and *Mamm* is the molecular mass of the organic cation exchanged on the surface. High resolution TGA also helps to ascertain the presence (or absence) of any excess surface modification molecules present in the interlayers which are not bound to the surface. These unbound molecules can be detected because of their low temperature thermal degradation as compared to the surface bound molecules. These unbound molecule present as pseudo bilayer on the surface of the layered silicate are important to be avoided as these molecules can cause unwanted interactions with the polymer systems as well as their low temperature degradation can cause reactions with the polymer matrices leading to the reduction of the polymer molecular weight [30,47,48]. Figure 12a shows the TGA thermograms of the fillers modified with various ammonium ions, the chemical structures of which are demonstrated in Figure 12b [45]. The absence of any low temperature degradation peak in the region of 200-300 °C in the thermograms (except 2C18) confirms the absence of any excess ammonium ions present in the interlayers. The thermograms corresponding the filler modified by dioctadecyldimethylammonium (2C18) shows a small peak which corresponds to some amount of organic matter degrading before the majority of the organic matter in the interlayer. This component corresponds to the presence of a small amount of excess modifier molecules present in the interlayers which are unbound to the surface. The removal of such excess molecules is more important when the fillers are to be used for high temperature compounding with the polymers. It has also been reported that the cleaning protocols required for the complete elimination of these excess modification molecules may require many cycles of rigorous washing before the surface of the clay is fully clean [47,49]. It is also important to note that the X-ray diffractograms are not able to discover the presence of the excess surface modification molecules as the basal plane spacing of the filler in the presence or absence of excess molecule is not affected. 

**Figure 13:**
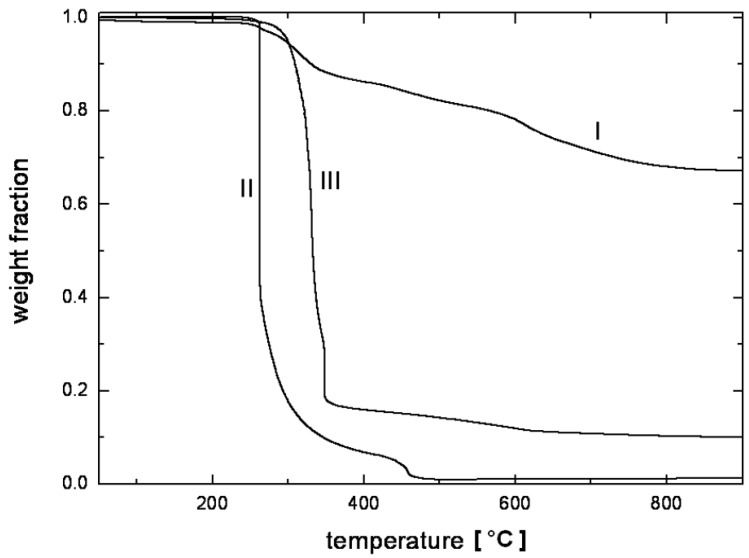
TGA thermograms of the (I) modified clay, (II) pure PP and (III) 4 vol% PP-OMMT composite. Reproduced from reference [50] with permission from Elsevier.

In the case of composites, TGA studies are required to analyze the synergism between the organic polymer and the inorganic filler phases. Figure 13 demonstrates such an analysis of the thermal performance of the nanocomposite generated from imidazolium modified filler incorporated into high molecular weight polypropylene [50]. The degradation behavior of the nanocomposite material clearly shows the synergism between the components of the composites as the onset of the degradation of the composites is better than both of the organic and inorganic constituents of the composite. It should also be noted that these composites contained only 4 vol% of the filler and the composites containing even lower amounts of filler also demonstrated similar synergetic improvement of the thermal behavior indicating that only a small amount of inorganic platelets is required to gain significantly improved properties in the nanocomposites. 

**Figure 14:**
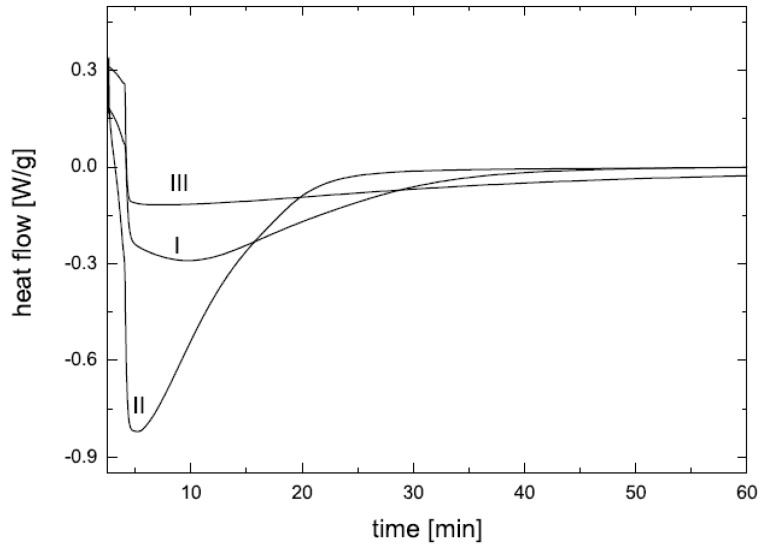
DSC thermograms indicating the effect of filler on curing at 70 °C; I: no filler, amine to epoxy mole ratio 0.3:1, II: 3.5 vol% 2C18-OMMT, amine to epoxy mole ratio 0.3:1 and III: 3.5 vol% 2C18-OMMT, amine to epoxy mole ratio 0.2:1 [9].

Differential scanning calorimetry is also used to gain further insights into the synthetic characteristics and microstructure of the nanocomposites [9]. The effects of the presence of filler on the overall epoxy curing process were studied as shown in Figure 14. Presence of dioctadecyldimethyl-ammonium modified filler led to a significant acceleration of the curing, as shown by curve II as compared to curve I without the presence of filler. It is because of the acidic nature of the ammonium ions which have been reported to catalyze the epoxy-amine polymerization reaction [51,52,53,54]. Although with quaternary ammonium ions, the extent of intergallery catalysis is reported to be lower as compared to primary, secondary and tertiary counterparts, but it can be still significant. Curve III indicates the curing process carried out at 70 °C at a reduced amine to epoxy mole ratio of 0.2:1. The slowing down of the curing process is clearly visible. 

## 4. Nanocomposite Preparation

There are four main routes for the synthesis of nanocomposites [4,39]: (a) template synthesis, (b) intercalation of polymer or prepolymer from solution, (c) *in-situ* intercalative polymerization and (d) melt intercalation. These techniques for the nanocomposite synthesis are reviewed in the following paragraphs:

### 4.1. Template Synthesis

This technique of template synthesis is not very widely used. In principle, it is totally opposite in nature than the other usual techniques. In the commonly used nanocomposite synthesis techniques polymer materials are generally synthesized in the presence of the silicate filler. However, in this technique, the inorganic material is synthesized in the presence of the polymer matrix. Double layer hydroxide based nanocomposites have been synthesized by using this route *in-situ* in an aqueous solution containing the polymer and the silicate building blocks [55,56]. The polymer aids the nucleation and growth of the inorganic host crystals and gets trapped within the layers as they grow. Though this technique presents high potential route for the dispersion of the layered silicate in the polymer matrices at nanometer scale, however it suffers from some drawbacks [4]. The synthesis process generally requires the use of high temperatures, which is detrimental for the polymer materials. The silicate materials generated by the self-assembly process have also the tendency to aggregate. 

### 4.2. Intercalation of Polymer or Prepolymer from Solution

**Figure 15:**
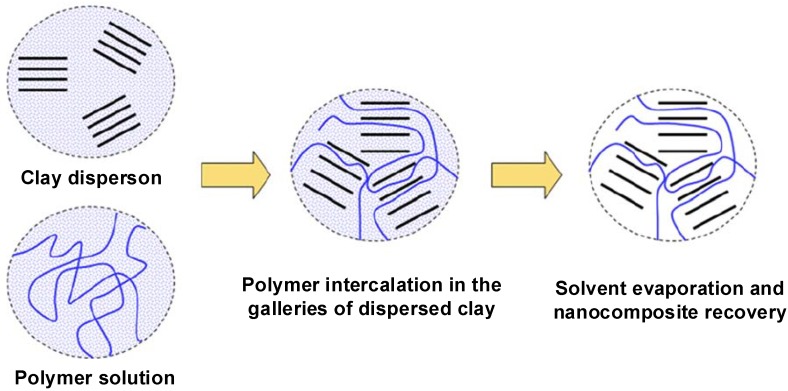
Schematic representation of polymer adsorption from solution. Reproduced from reference [4] with permission from Elsevier.

In this mode of nanocomposite synthesis, the organically modified silicate is dispersed in a solvent in which the polymer is also soluble. It is well known that such layered silicates, owing to the weak forces that stack the layers together can be easily dispersed in an adequate solvent. The polymer then adsorbs onto the delaminated sheets and when the solvent is evaporated (or the mixture precipitated), the sheets reassemble, sandwiching the polymer to form, in the best case, an ordered multilayer structure. Under this heading we also include the nanocomposites obtained through emulsion polymerization where the layered silicate is dispersed in the aqueous phase [39]. The use of solvent though leads to the generation of intercalated nanocomposites, however, this approach is not environmentally friendly owing to the use of large amounts of solvent. The schematic of the process is shown in Figure 15 [4]. The polymer chains lose entropy owing to diffusion inside the silicate interlayers, however, such a process is still thermodynamically viable, owing to the gain in the entropy by the solvent molecules due to desorption from the silicate interlayers [57,58].

The technique is mostly used for the intercalation of the water soluble polymers like poly(vinyl alcohol), poly(ethylene oxide), poly(acrylic acid), poly(vinlypyrrolidone) etc. [59,60,61,62,63]. Later on, the use of this technique was also undertaken in organic solvents for polymers that are non-soluble in water. High density polyethylene was used as polymer matrix which was dissolved in a mixture of xylene and benzonitrile [64]. Biodegradable nanocomposites using poly(lactic acid) as polymer matrix were also reported using dichloromethane as solvent [65]. The exfoliated and intercalated nanocomposites were obtained depending on the type of organically modified montmorillonite. 

Some polymers like poly(imides) do not dissolve in organic solvents, therefore, in such cases, prepolymer or polymer precursor is intercalated in the silicate interlayers which is subsequently converted in the polymer matrix [39]. Preparation of poly(imide) montmorillonite nanocomposites by mixing poly(amic acid) in dimethylacetamide in the presence of modified montmorillonite was reported by Toyota researchers [12]. The montmorillonite was exchanged with dodecylammonium hydrochloride. The heat treatment of the resulting hybrid led to the generation of the exfoliated nanocomposites as the filler diffraction peaks in the XRD diffractograms were absent. Conjugated polymers have also been reported to be intercalated in the filler interlayers by the similar precursor intercalation approach. To achieve poly(p-phenylenevinylene) montmorillonite composites, poly(p-xylenylene dimethylsulfonium bromide) precursor was intercalated into the layered silicates [66,67]. The precursor was then transformed into polymer by base mediated elimination of dimethylsulfide and HBr.

Emulsion polymerization of monomers in the presence of the layered silicates has also been reported to generate intercalated and exfoliated nanocomposites. Most widely used monomers haven been methyl methacrylate and styrene [68,69]. The monomer is suspended in water along with varying amounts of silicates in the presence of emulsifier. The monomer is then polymerized leading to the generation of the nanocomposites with a part of silicate embedded inside the polymer particles and a part adsorbed on the surface of the particles. 

### 4.3. In-Situ Intercalative Polymerization

The *in-situ* intercalation mode of polymerization was the method reported by Toyota researchers which led to the exponential growth in nanocomposites research. In this mode of polymerization, the layered silicate mineral is swollen in monomer. The monomer. being of low molecular weight. can also diffuse easily into the interlayers thereby swelling the interlayers. On initiation of the reaction, the monomer present in and out of the interlayers polymerizes to generate nanocomposites in which the layered silicate platelets are delaminated to the nanometer level. However, a control of the polymerization in and out of the layers is required in order to achieve high extents of filler exfoliation. 

**Figure 16:**
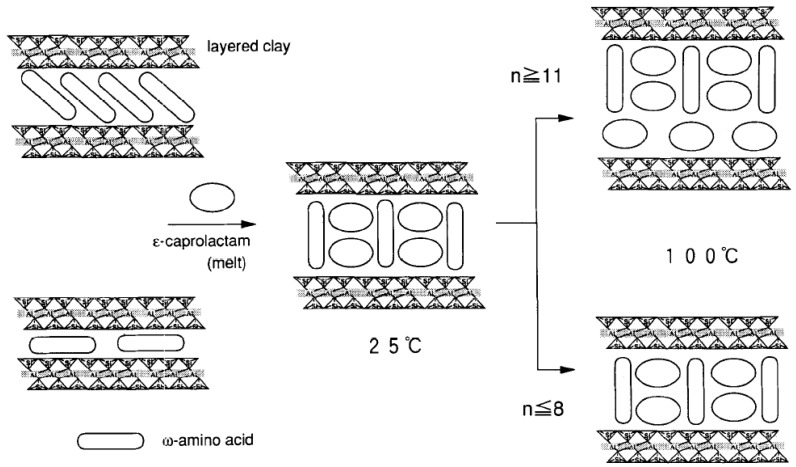
Intercalation of the modified montmorillonite with caprolactam. Reproduced from reference [70] with permission from Elsevier.

Toyota researchers reported the modification of the montmorillonite with amino acids of different chain lengths which were subsequently swollen by caprolactam. The schematic of such a process is shown in Figure 16 [70]. The ring opening polymerization of carpolactam was then initiated to obtain nylon 6 nanocomposites. The importance of the chain length in the modification was also demonstrated as large amount of monomer could be intercalated in the interlayers when large chain lengths were used for ion exchange on the surface of the silicate. In a typical synthesis step, montmorillonite modified with 12-aminolauric acid was mixed with caprolactam followed by heating of the mixture at 250–270 °C for 48 h for caprolactam polymerization. Modified montmorillonite was used as a catalyst. 6-aminocaproic acid was also used a polymerization accelerator when the relative amount of modified montmorillonite in the mixture was less than 8 wt%. From the characterization of the composite morphology by XRD and TEM, it was observed for less then 15 wt% of the filler, exfoliated nanocomposite were obtained,, whereas, for filler amounts in the range from15 to 70 wt%, intercalated nanocomposites were obtained. It was also further demonstrated by the authors that the modification of the filler is not necessary to obtain the polyamide nanocomposites [71]. In the presence of hydrochloric acid, the monomer was able to intercalate the sodium montmorillonite platelets in water. The basal plane spacing of the pristine filler was observed to increase from 10 Å to 15.1 Å owing to the intercalation of caprolactam. Exfoliated nanocomposites were obtained after the ring opening polymerization of the monomer. 

**Figure 17:**
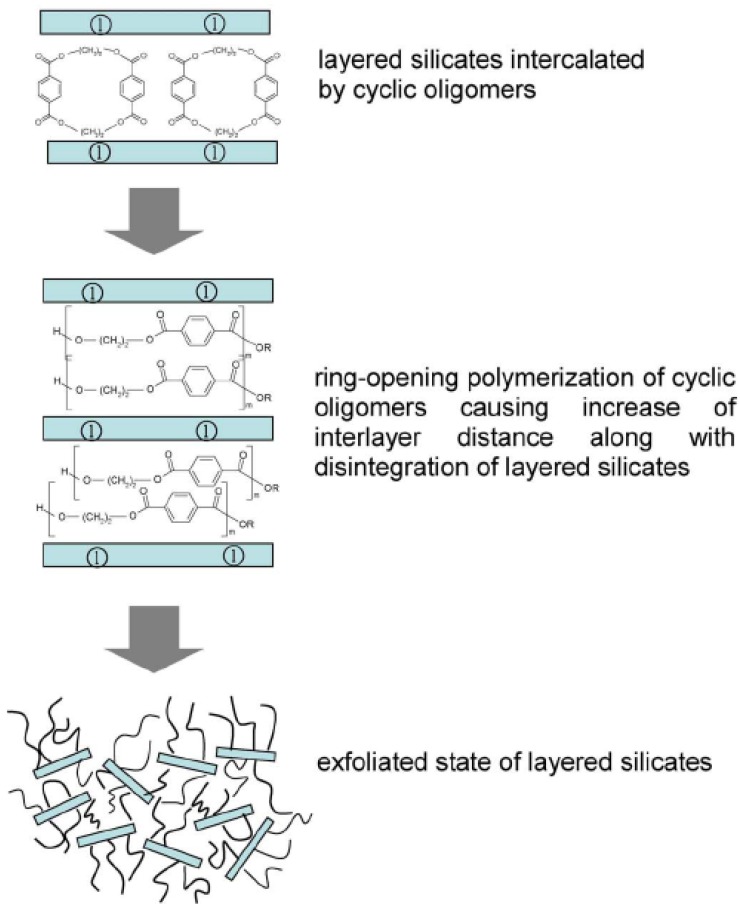
Ring opening polymerization of cyclic oligomers to generate polymer nanocomposites. 1 in the figure represents the layered silicate material. Reproduced from reference [72] by permission from Elsevier.

The *in-situ* polymerization technique has also been used for other polymer systems. In one such study [72], the authors presented the problems associated with the generation of PET nanocomposites. Direct condensation reactions of diol and diacid in presence of clay led to the generation of low molecular weight polymers, whereas the synthesis of nanocomposites by melt intercalation approach leads to only small extent of polymer intercalation in the interlayers. Ring opening polymerization of ethylene terepthalate cyclic oligomers with organically modified montmorillonites was suggested as an alternative to achieve exfoliated PET nanocomposites as shown in Figure 17. Owing to the low molecular weight, these cyclic monomers can be easily intercalated in the interlayers leading to large extent of interlayer polymerization subsequently.

**Figure 18:**
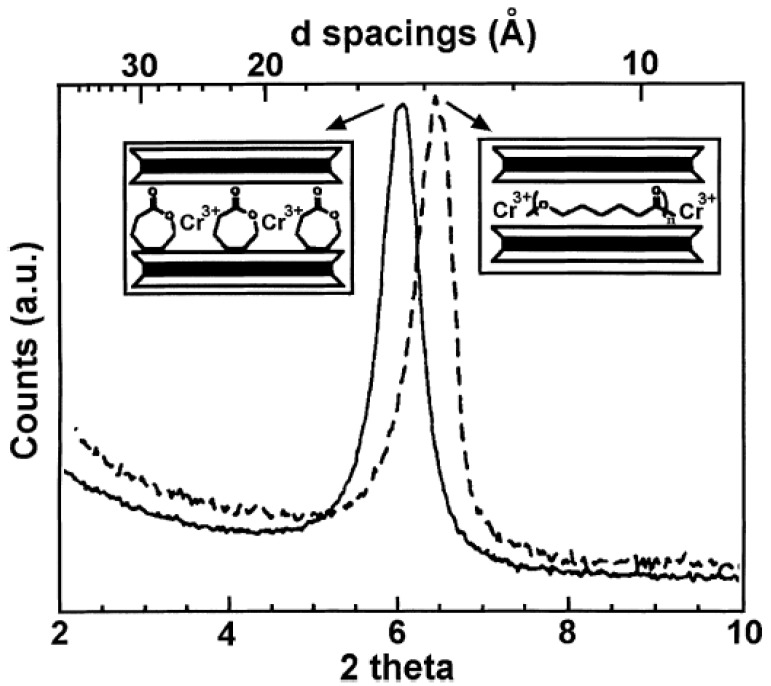
X-ray diffractograms of the intercalated filler (solid line) and the poly(caprolactone) based nanocomposite material (dashed line). Reproduced from reference [73] with permission from The American Chemical Society.

Cr^3+^ modified fluorohectorite has also been used for the polymerization of caprolactone inside the interlayers [73]. The polymerization was carried out at 100 °C for 48 h. The intercalation of the monomer inside the silicate interlayers was followed by X-ray diffraction. An increase of basal plane spacing from 12.8 Å for the un-intercalated filler to 14.6 Å for the intercalated filler was observed. Due to the dimensional change during the ring opening polymerization of caprolactone, a final basal plane spacing of 13.7 Å was observed for the nanocomposite, as shown in Figure 18. The nanocomposite basal plane spacing of 13.7 Å was reported to correlate well with the sum of interlayer spacing of 9.6 Å for the pristine silicate and 4 Å for the inter-chain distance in the crystal structure of poly(caprolactone). 

Use of the polymerization filling technique has also been reported for the synthesis of polyolefin nanocomposites by *in-situ* polymerization [74]. In this technique, a Zieler-Natta or any other co-ordination catalyst is anchored to the surface of the layered silicates. This then can be directly used or the polymerization of olefins like ethylene, propylene etc. from the surface of the silicate. Here the immobilization of the catalyst does not require any cation exchange on the surface of the silicate, rather the immobilization is carried out by the electrostatic interactions of the catalytic materials with the MAO initially anchored to the filler surface. 

Polymerizations are generally carried out under dilute conditions. In one such instance, when the polymerization of ethylene was carried out in the absence of any chain transfer agent, ultra high molecular weight polyethylene-silicate nanocomposite was achieved, which could not be processed further. However, by adding hydrogen to the system, the molecular weight of the polymer matrix could be reduced and the processability also improved [74]. Similarly, the use of palladium based complex and synthetic fluorohectorite as polymerization catalyst and inorganic component for the generation of polyethylene nanocomposites have been reported [75]. Titanium based Ziegler Natta catalysts were similarly immobilized in the inner surfaces of montmorillonites and organic salts with hydroxyl groups for the modification of montmorillonite were used [76]. The hydroxyl groups acted as reactive sites for anchoring catalyst between the clay layers. Heinemaan *et al.* [77] also reported the polymerization of ethylene in the presence of modified layered silicates. Surface modifications like dimethyldistearylammonium and dimethylbenzylstearyl-ammonium were used for the study. The composites were also prepared with the non-modified fillers. It was reported that nanocomposites were only observed when the modified clays were used, whereas in the case of non-modified fillers, only microcomposites were formed. 

Vinylbenzyltrimethylammonium modified montmorillonite intercalates, which are able to swell and disperse in organic solvents were prepared by a cation exchange process [78]. The resulting vinyl monomer-montmorillonite materials were confirmed by the combination of various techniques like X-ray diffraction (XRD), elemental analysis and infrared absorption spectra. The vinyl monomer groups were copolymerized with externally added styrene which resulted in grafted polystyrene montmorillonite materials. The effect of montmorillonite amounts on the formed polystyrene was determined by extraction with organic solvents, which showed an increase in the grafted polymer formed (0.84-2.94 g/g MMT), and a decrease in the free polystyrene with increasing amounts of montmorillonite. Similarly, a polymerizable cationic surfactant, vinylbenzyldimethyldodecyl-ammonium chloride (VDAC) with terminal monomer moiety was synthesized and was ion exchanged in an aqueous medium on the clay surface to generate functional organophilic MMT [79]. The modified clay was swollen with styrene monomer and subsequent free radical polymerization of styrene with azo bis(*iso*-butyronitrile) (AIBN) as initiator led to the generation of polystyrene–clay nanocomposites. Exfoliation of MMT in polystyrene matrix was achieved as revealed by X-ray diffraction (XRD) and transmission electron microscopy (TEM). The exfoliated nanocomposites had higher dynamic modulus and higher decomposition temperature than pure polystyrene. In an another study, a free-radical initiator 2,2'-azobis(isobutyramidine hydrochloride) (AIBA) was attached by ion exchange to the surface of an ultrahigh specific surface area muscovite mica powder [80]. The modified filler was dispersed in styrene followed by free radical polymerization. As the initiator was attached on the surface, the grafting was therefore expected to occur from the clay surface, but it was found that grafting proceeded by an unexpected mechanism. Instead of propagation of free radicals from the surface into the bulk monomer (growth from the surface), grafting took place via attack of growing chains thermally-initiated in the monomer on disproportionation products of AIBA attached to the surface (growth to the surface). The bound polymer consisted predominantly of high molecular weight chains bound to a very small fraction of the surface ion-exchange sites. Organic modifications with di-vinyl groups (*N*-methyl-*N,N*-di(vinylbenzyl)octadecylammonium chloride) were also reported and exfoliated composites of both polystyrene and poly(methyl methacrylate) were achieved [81]. 

Cation bearing monocationic initiators [4-(*tert*-butyldioxy)butyl]trimethylammonium bromide (I-4), [4-(*tert*-butyldioxy)hexyl]trimethylammonium bromide (I-6), and [4-*tert*-butyldioxy)decyl]-trimethylammonium bromide (I-10) were prepared and ion exchanged on the surface of ultrahigh specific area delaminated mica [82,83]. The affinity of the initiator to attach to clay surface was reported to be dependant on the number of methylene groups in the chain. Therefore, a large part of the initiator I-4 was removed from the surface upon washing, while for I-6 and I-10, most of the adsorbed initiator remained on the surface. Poly(styrene) grafted to mica surfaces was obtained on the polymerization of styrene in the presence of initiator modified mica. The bound polymer was formed following a first order reaction kinetics, which was markedly different from the polymerization of styrene in the presence of mica modified with AIBA, where the polymerization followed zero-order kinetics. Presence of the polymer chains on the surface of the mineral could be confirmed by microscope, though a large proportion of the formed polymer was in solution i.e. unattached to the clay surface owing to the release of an initiating moiety per initiator molecule into the solution. Scanning electron microscopy analysis confirmed the presence of polystyrene molecular droplets on the inorganic surface, the density of which could be varied by varying the polymerization time. The droplets could be made to coalesce into thin films by increasing the grafting density, by heating, or by solvent treatment.

**Figure 19:**
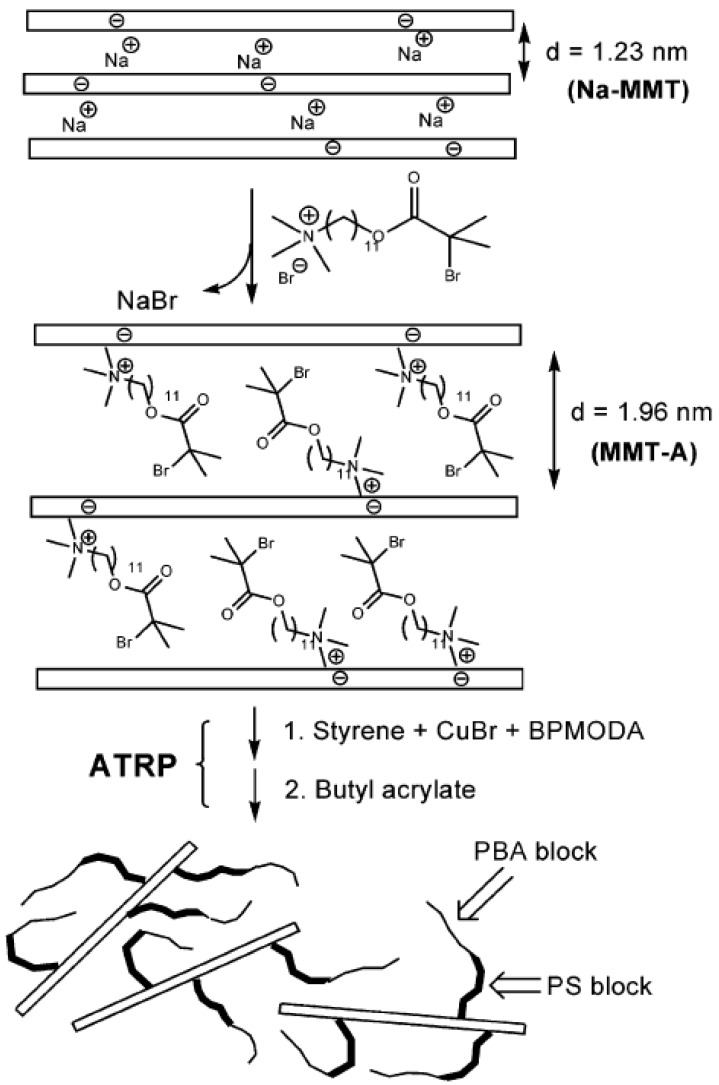
Synthesis of block copolymer layered silicate nanocomposites by using atom transfer radical polymerization. Reproduced from reference [84] by permission from The American Chemical Society.

Zhao *et al.* reported the generation of block copolymer of poly(styrene-block-butylacrylate) (PSBA) grafted from the clay surface by using ATRP approach [84] (Figure 19). *N,N*-bis(2-pyridiyl-methyl)octadecylamine (BPMODA) was used as amine and ATRP initiator was 11’-(*N,N,N*-trimethylammonium bromide)undecyl-2-bromo-2-methyl propionate. The polymerization of styrene was carried out at 110 °C with the ATRP initiator modified clay along with BPMODA and CuBr. Butyl acrylate was subsequently polymerized to generate block polymer grafted from clay surface. The study successfully confirmed the capability of this approach to control the nature of clay surface as required. Transmission electron microscopy (TEM) analysis showed the existence of both intercalated and exfoliated structures in the nanocomposite. It was further reported the use of this technology in the generation of nanocomposites using styrene, methyl methacrylate and butyl acrylate as monomers [85]. An ATRP initiator, consisting of a quaternary ammonium salt moiety and a 2-bromo-2-methyl propionate moiety, 11’-(*N,N,N*-trimethylammonium bromide)undecyl-2-bromo-2-methyl propionate was exchanged onto the clay surface. Catalysts used were Cu(I)X/*N,N*-bis(2-pyridiylmethyl)octa-decylamine, Cu(I)X/*N,N,N’,N’,N’’*-pentamethyldiethylenetriamine, or Cu(I)X/1,1,4,7,10,10-hexa-methyltriethylenetetramine (X = Br or Cl). Polymers with high molecular weight and lower polydispersities could be successfully achieved. With TEM and X-ray diffraction, it was observed that the polystyrene nanocomposites contained a mixed morphology with intercalated and exfoliated clay platelets, whereas the poly(methyl methacrylate) nanocomposites were significantly exfoliated. One could expect that PMMA being polar results in more exfoliated morphologies. However, it was suggested by the authors that greater polarity does not guarantee greater mixing, as the final microstructure is a resultant effect of factors such as the temperature, solvent, silicate type, and modification etc.

**Figure 20:**
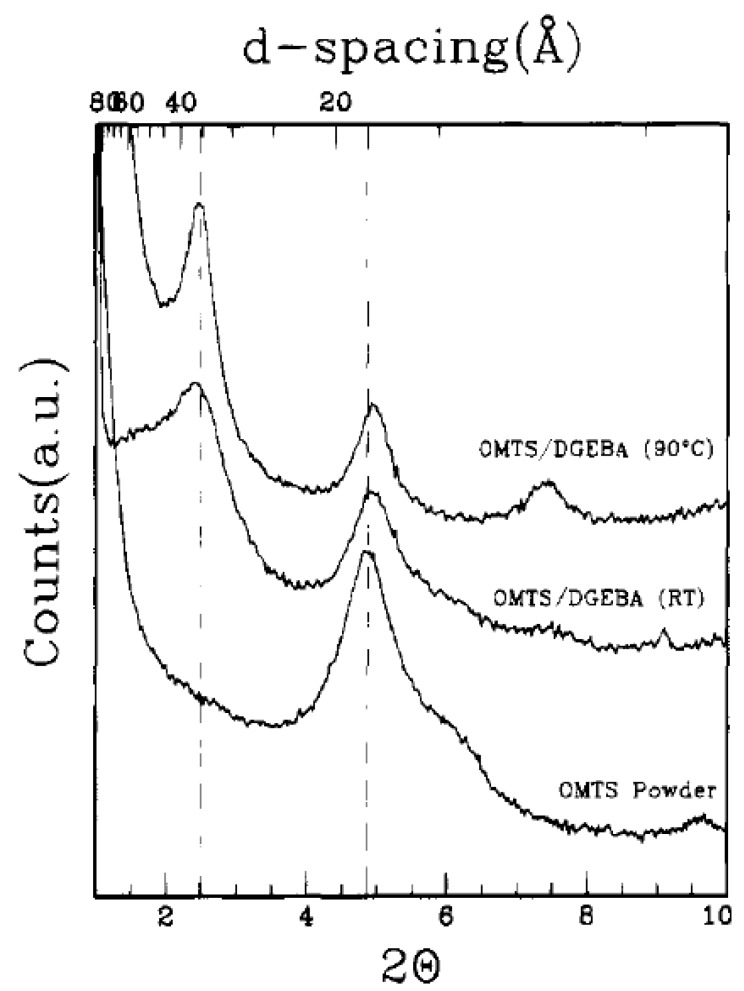
X-ray diffractograms of the modified filler and the mixture of epoxy prepolymer with filler at room temperature and at 90 °C. Reproduced from reference [86] with permission from The American Chemical Society.

**Figure 21:**
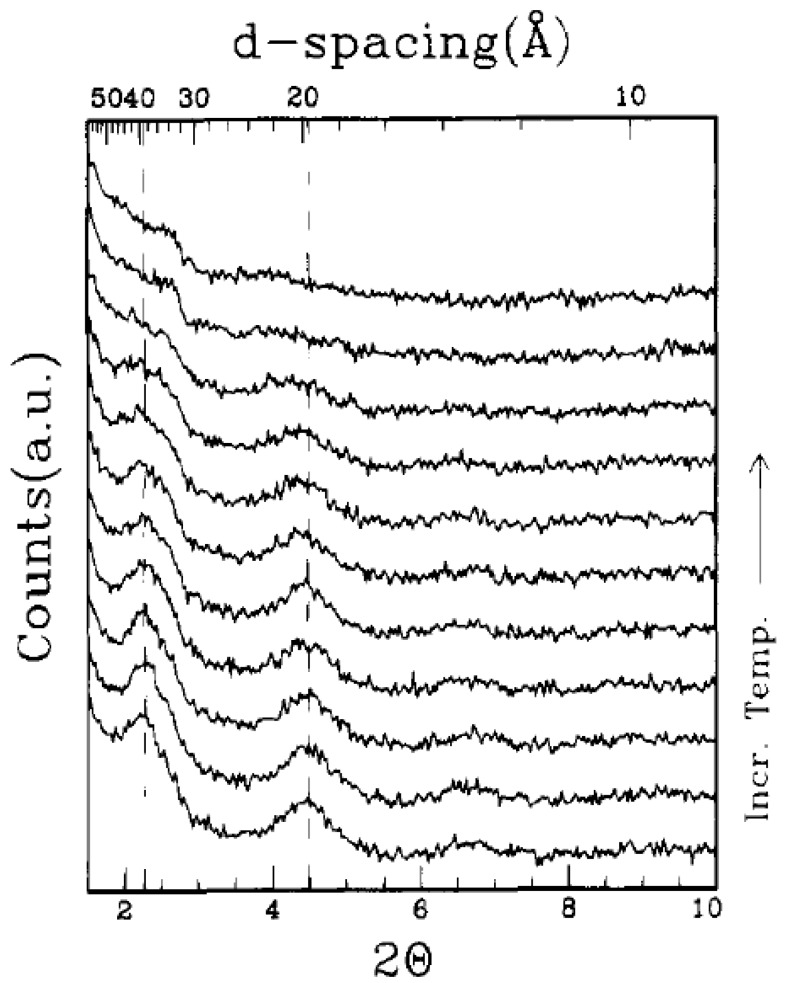
X-ray diffractograms of filler/DGEBA/benzyldimethlyamine system at different temperatures from 20 °C to 150 °C. Reproduced from reference [86] with permission from American Chemical Society.

This technique is also the most promising method for the generation of thermoset polymer nanocomposites. The technology was first presented by Messersmith and Giannelis [86]. They studied the synthesis of epoxy nanocomposites by using different curing agents and curing conditions. It was observed that the montmorillonite modified with bis(2-hydroxyethyl)methyl halogenated tallow ammonium ions was readily dispersible in the diglycidyl ether of bisphenol A (DGEBA). The layer spacing was observed to increase when filler was added with DGEBA confirming the intercalation of DGEBA in the filler interlayers as shown in Figure 20. The intercalation was also observed to increase when the temperature was raised from room temperature to 90 °C. The DGEBA prepolymer molecules owing to their low molecular weight as well as polar nature can intercalate efficiently in the partially polar interlayers of silicates thus expanding them further apart. Figure 21 also shows the generation of exfoliated morphology in the silicate/DGEBA/benzyldimethlyamine system [39,86]. At room temperate, a mix of both of intercalated and unintercalated silicate platelets is present. At increasing temperature, the diffraction peaks were observed to disappear indicating delamination occurred during the heating. The nature of the curing agent also significantly affected the generated morphology of the composites. When diamines were used as curing agents, only intercalated composites were obtained. However, when other curing agents like anhydrides, benzyldimethlyamine etc. were used, exfoliated nanocomposites were obtained. In the case of diamines as curing agents, the authors suggested that the bridging of the silicate layers by the bifunctional amine molecules may hinder their exfoliation.

Epoxy nanocomposites using different surface modifications benzyldibutyl(2-hydroxyethyl)-ammonium chloride (Bz1OH), benzyl bis(2-hydroxyethyl)butylammonium chloride (Bz2OH), benzyltriethanolammonium chloride (Bz3OH), benzyl(2-hydroxyethyl)methyloctadecyl ammonium chloride (BzC18OH), dioctadecyldimethyl ammonium chloride (2C18) and benzyldimethylhexadecyl-ammonium chloride (BzC16) were reported [45]. 

**Figure 22:**
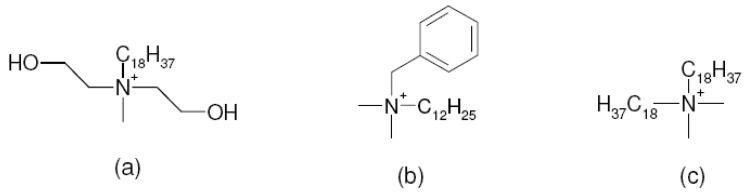
Chemical structures of the modifications exchanges on the filler surface for the generation of polyurethane nanocomposites [9,87].

The compatibility of the filler surface modification with the epoxy prepolymer was reported to significantly affect the final morphology of the composites. The initial basal plane spacing of the modified filler was not the deciding factor for the achievement of exfoliated composites. Using benzyldibutyl(2-hydroxyethyl)ammonium as surface modification though did not increase the basal plane spacing of the filler much, but its better polarity match with the prepolymer molecules led to the generation of the exfoliated nanocomposites after curing. On the other hand, modifications like dioctadecyldimethyl-ammonium chloride (2C18) and benzyldimethylhexa-decylammonium chloride (BzC16) resulted in modified fillers with significantly increased basal plane spacing values, however, led to maximum intercalated nanocomposites owing to the poor compatibility of surface modification with the epoxy prepolymer. The mole ratio of the crosslinker to the epoxy prepolymer and the polymerization temperature were crucial to be optimized to achieve a balance between the polymerization in and out of the interlayers. Polyurethane nanocomposites have also been studied in the similar fashion [87]. The modification with hydroxyl groups in the chemical structure (Figure 22) matched better with the polyurethane matrix thus generating exfoliated nanocomposites with much superior properties. On the other hand, non-polar surface modifications (Figure 22c) on the filler surface resulted in decreased resistance to oxygen permeation due to the generation of the micro voids at the organic inorganic interface owing to the mismatch between the polar polymer matrix and the non-polar surface modification. Other polymer systems have also been extensively studied using in situ polymerization approach [88,89,90,91,92].

### 4.4. Melt Intercalation

Melt intercalation has developed into one of the most attractive methods for the commercial generation of polymer nanocomposites. In this approach, the polymer is first melted at high temperature and the filler is then blended with the polymer melt at high temperature under shear. This technique has an advantage that no solvent is required for the nanocomposite synthesis. The polymer can intercalate between the interlayers if the silicate surface is modified in a way that the electrostatic forces holding the platelets together are very weak. The use of high temperature required to achieve a homogenous mix can however lead to occasional degradation of the surface modification and polymer thus requiring care during the compounding process. As this method is environmentally friendly and does not require the usage of large amounts of solvents, and also owing to its simplicity and economic viability, the melt intercalation method has been widely used for the synthesis of polymer nanocomposites with a large number of polymer materials [93,94,95,96,97,98,99,100]. 

A lattice model theory has been suggested by Vaia and Giannelis to explain the thermodynamics that drives the intercalation of molten polymer chains inside a modified layered silicate [101]. It was postulated that the outcome of polymer intercalation is influenced by interplay of entropic and enthalpic factors. When the polymer chains intercalate the silicate layers, it results into a decrease in the overall entropy of the macromolecular chains. This decrease in the entropy of the chains may be compensated by the increase in conformational freedom of the tethered alkyl surfactant chains as the inorganic layers separate, due to the less confined environment. As small increases in the gallery spacing do not influence strongly the total entropy change, intercalation is rather driven by the changes in total enthalpy. The enthalpy of mixing was classified in two components: apolar interactions which are generally unfavorable and arise from interaction between polymer and surfactant aliphatic (apolar) chains, and polar interactions which originate from the polar silicates interacting with the polymer chains [39]. The enthalpy of mixing can thus be rendered favorable by maximizing the magnitude and number of favorable polymer-surface interactions while minimizing the magnitude and number of unfavorable apolar interactions between the polymer and the aliphatic chains introduced along the modified layer surfaces. 

For polar polymer matrices like polyamides, the interactions of the polymer materials with the polar silicate surface drive the polymer chains inside the clay layers. However, the situation is quite different when polyolefins are involved. Theoretical studies implying the use of self consistent field (SCF) models have also predicted the self-consistent potential or tendency of exfoliation to be a function of the grafting density of the tethered surfactants and the Flory Huggins interaction parameter or χ values [102,103]. Favorable enthalpic interactions between the OMMT and the polymers can overwhelm the entropic losses and lead to effective intermixing of polymer and clay. For the mixtures of long chain homopolymers with the organically modified clays to be thermodynamically stable, χ must be less than zero. Even then such structures were predicted to exhibit an intercalated morphology without exfoliation for polyolefins. Increasing the length of the tethered surfactants improved the thermodynamic state of the system as more distance generated among the clay layers helped in bringing down the effective interactions between the clay sheets. For a given density of alkyl chains on the surface, long chains were predicted to form a more homogenous phase than the short ones [104]. Thus, even in the absence of any attractive interaction between the long polymer chains and the surfactant molecules (χ = 0) i.e., at theta conditions, the increase in the d-spacing by incorporating longer surfactant chains can help in achieving more delamination. Grafting density also was predicted to bear a significant influence on the final morphology of the composite as too loose and too packed clay platelets were found unfavorable to result in effective mixing [105]. Autophobicity and subsequent dewetting are another important phenomenon reported when chemically identical chains as the polymer were grafted on the polymer surface [106]. Thus, proper optimization of the organic monolayer structure in combination with the mechanical shear is of utmost requirement to achieve maximum exfoliation. 

To improve attractive interactions between the apolar polymer and polar silicate surfaces and to decrease repulsive forces between the polymer and the surface modification, partial polarization of the polymer is also achieved by the use of compatibilizers like polypropylene grafted with maleic anhydride (PP-g-MA). In fact, almost all of the reported polypropylene nanocomposites incorporating organically modified montmorillonite (OMMT) prepared by melt compounding approach use low molecular weight PP-g-MA compatibilizer or non-ionic surfactant to achieve better compatibility between the polar clay interlayers and apolar polypropylene matrix, thus, to achieve clay exfoliation [107,108,109,110,111,112,113,114,115,116,117]. OMMT exfoliation and the properties of the composites were found to be independent of the molecular weight of the compatibilizer, but were strongly dependant on the weight fraction of the compatibilizer and the extent of grafting of maleic anhydride. In addition, Reichert *et al.* also reported the higher extent of exfoliation for octadecyl chains attached to the silicate surface as compared to the smaller alkyl chains [111] in the presence of the compatibilizer. The majority of the studies on polypropylene nanocomposites bring home the conclusion that the use of the maleic anhydride modified polypropylene as compatibilizer favors intercalation of the polymer between the clay layers, but occasionally on the expense of the mechanical properties. Kato *et al.* reported the synthesis of maleic anhydride grafted polypropylene-organo clay hybrids using protonated octadecylamine as surface modification [107]. PP-g-MA with molecular weight (M_w_) of 30,000 g.mol^-1^ and acid value of 52 mg KOH·g^-1^ was observed to have better intercalation and higher d-spacing than the PP-g-MA with a molecular weight (M_w_) of 12,000 and an acid value of 7 mg KOH·g^-1^, in which no intercalation was noticed. Hasegawa *et al.* also reported X-ray silent nanocomposites of PP-g-MA (MA content 0.2 wt% and M_w_ of 210,000) with C18-OM [118]. These hybrids were prepared as masterbatches which were diluted with PP to give the final composites in the hope that this will lead to an exfoliated structure. However, the PP chains did not diffuse in the interlayers and the Young’s modulus of the PP nanocomposites increased by 20% only and the elongation at break and tensile strength decreased [110]. Almost exfoliated PP nanocomposites (C18-OM) were obtained when PP-g-MA with an acid value of 26 mgKOH·g^-1^ and M_w_ of 40,000 was used in stead of PP-g-MA of acid value of 52 mgKOH·g^-1^ and M_w_ of 30,000 [108]. A 1.6-1.7-fold increase of storage modulus was reported for both composites. 

**Figure 23:**
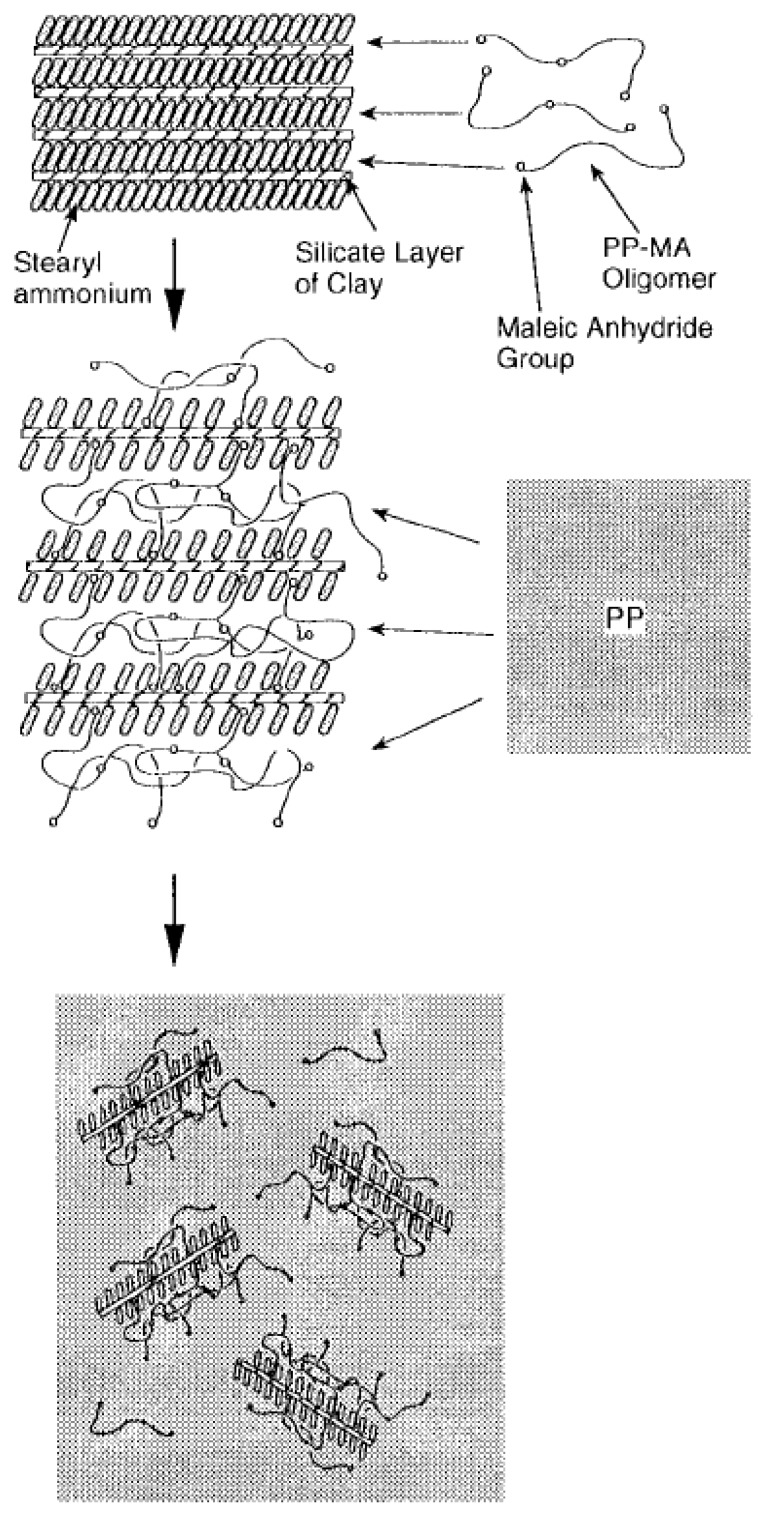
Schematic of polymer intercalation in the silicates in the presence of PP-g-MA. Reproduced from reference [108] with permission from The American Chemical Society.

Reichert *et al.* reported that similar C18-OM polypropylene composites with PP-g-MA compatibilizers have higher extent of dispersion, but lower impact strength [111]. Oya in contrast reported an increase in the notched izod impact strength from 2 to 3.4 kJ.m^-2^ for a 3 wt% C18-PP composite with PP-g-MA compatibilizer [114,119]. Zhang *et al.* also reported an increase in the impact strength from 9.4 kJ.m^-2^ for the pristine PP to 25.9 kJ.m^-2^ for the PP composite compatibilized with PP-g-MA at 2C18 filler loading of only 0.1 wt% [113]. Manias *et al.* reported nanocomposites using the fluorosilane compounds as compatibilizer [112]. The young’s modulus increased by 25% and only a small decrease in the strain at break was observed at 2 vol% filler content. The process of polymer intercalation in the presence of PP-g-MA is also schematically demonstrated in Figure 23 [108]. Other similar studies have also been reported [120,121].

**Figure 24:**
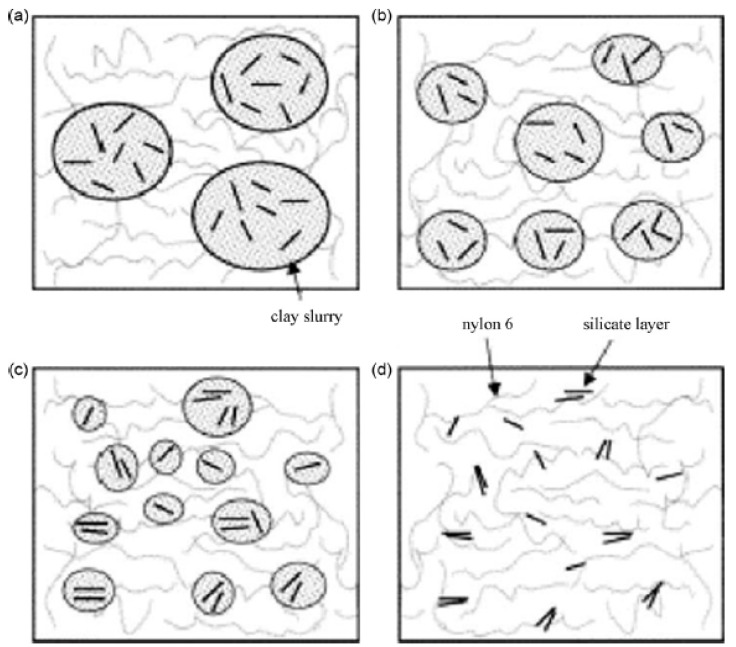
Dispersion of aqueous slurry of sodium montmorillonite in polyamide 6. Reproduced from reference [123] with permission from Elsevier.

Zheng *et al.* reported a functional oligomer as the surface modification of montmorillonite [122]. The oligomer was prepared from maleic anhydride, styrene and vinylbenzyltrimethylammonium chloride and the polystyrene nanocomposites were prepared by melt blending. A novel compounding process was also reported by Hasegawa *et al.* [123] for the preparation of polyamide nanocomposites. Sodium montmorillonite slurry in water was blended with polyamide 6 in the compounder followed by the removal of water. Thus, no surface modification was required to organophilize the surface of the montmorillonite. The process is demonstrated in Figure 24. The process of dispersion of the silicate platelets is quite different as compared to the conventional process. In this process, the exfoliated platelets are directly fixed in the polymer matrix without aggregation, whereas in the conventional process, the polymer chains diffuse into the silicate interlayers.

**Figure 25:**
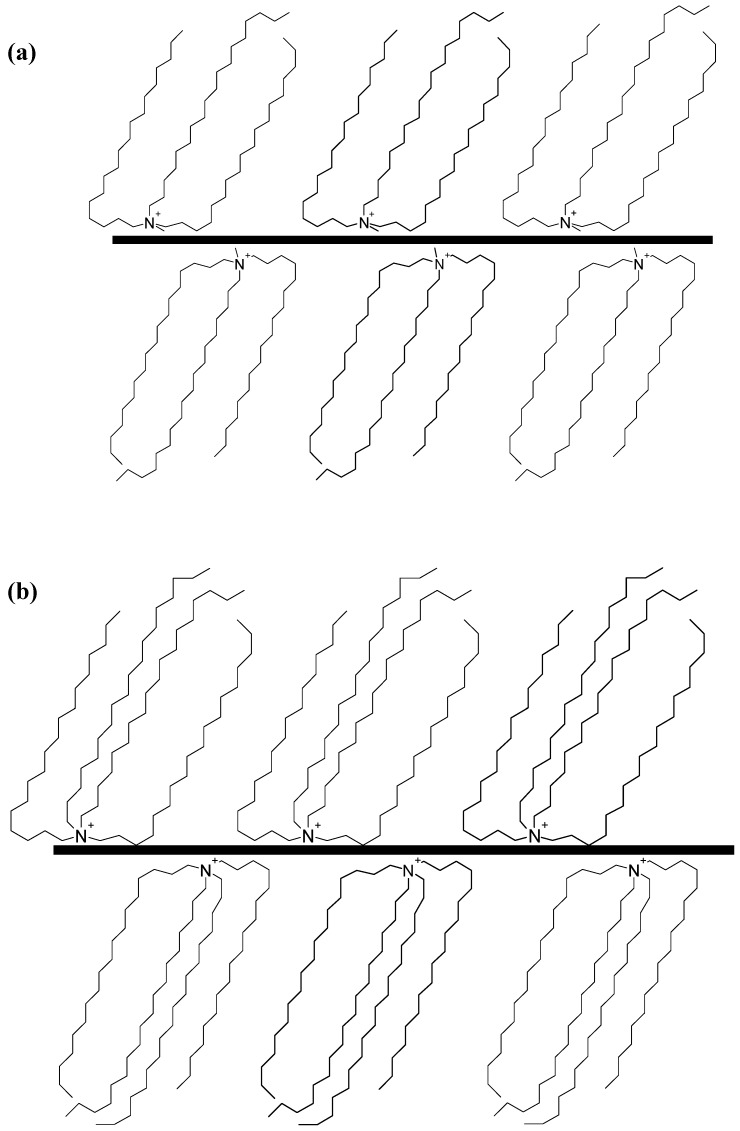
Filler platelets modified with (a) trioctadecylmethylammonium and (b) tetraoctadecylammonium. Reproduced from reference [124].

Optimization of the filler surface modification has been developed in the recent years to circumvent the limitations of the compatibilizers. The optimization includes the improvement in the basal plane spacing of the filler to as high value as possible. It has been mentioned in the earlier section that for the polar polymers, it is more the interfacial interactions which are of more importance than the filler basal spacing. However, in the case of non-polar polymer matrices, there is no interaction present at the interface and in such cases increased basal plane spacing of the filler owing to advanced modification can be very beneficial for its exfoliation in the polymer matrix. Increased basal spacing leads to reduced forces of attraction holding the platelets together and thus these loosely held platelets are more susceptible to exfoliation when sheared in the compounder along with the polymer matrix. The most obvious way to achieve higher basal plane spacing in the modified fillers is by increasing the chain density in the ammonium modifications. By increasing the chain density of the ammonium modifications, the cross-sectional area of the ammonium group increases. Thus, it occupies more space on the filler surface leading to more crowding of the surface modification on the filler surface which subsequently leads to more vertical positioning of the surface modification molecules than the case when the chain density in the modification molecules is low. This straighter positioning of the modification molecules leads to higher basal plane spacing and subsequent reduction in the electrostatic forces of interaction holding the platelets together. Figure 25 shows the filler platelets modified with trioctadecylmethylammonium and tetraoctadecylammonium modifications [124]. These surface modifications increase the basal plane spacing of the filler materials to more than 3 nm as compared to 0.9 or 1 nm for the unmodified platelets [49,125,126]. If higher cation exchange capacity montmorillonite is used, the basal plane spacing can be further increased. The high cation exchange capacity filler has higher charge density on the filler surface which means more amount of organic matter can be ionically exchanged on the clay surface owing to large number of charges per unit area. This then leads to increase in the basal plane spacing and subsequently filler delamination is improved. The polyethylene and polypropylene nanocomposites were synthesized with the montmorillonites modified as in Figure 25 and partial exfoliation of the filler could be achieved even without the use of compatibilizer [49,125,126]. The composite properties were also significantly improved. Table 1 shows the basal plane spacing values of montmorillonites modified with various surface modifications [127].

**Figure 26:**
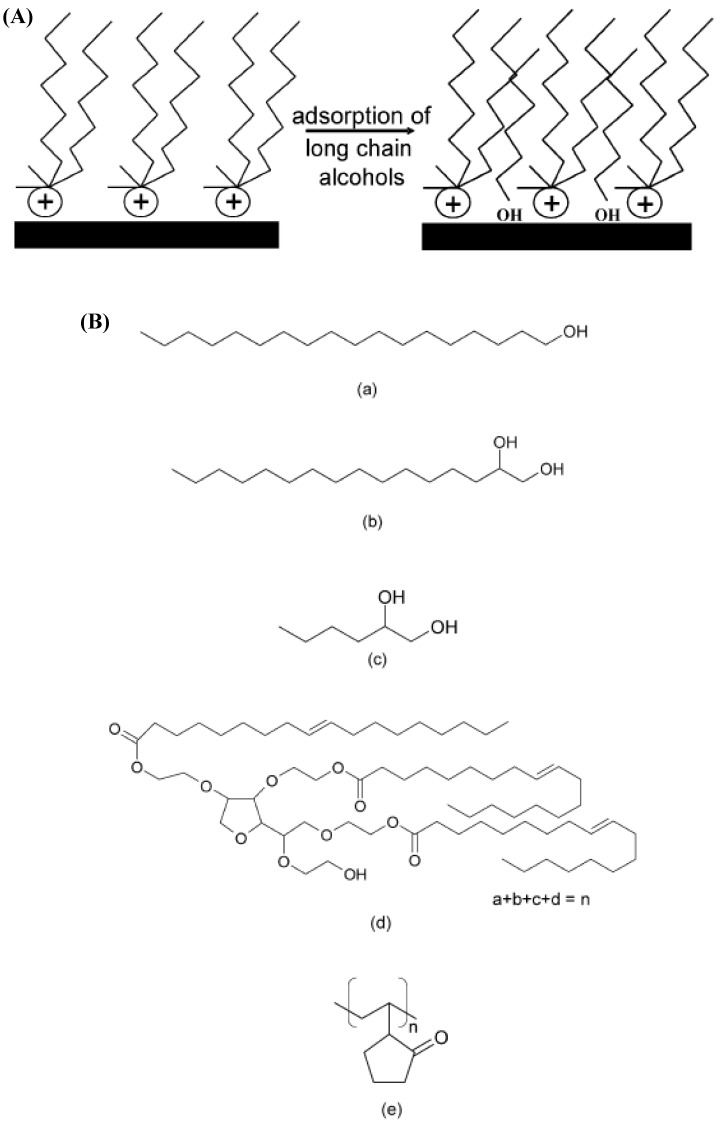
(A) Representation of physical adsorption process and (B) chemical structures of the adsorbents used for adsorption on the clay surface: (a) 1-octadecanol, (b) 1,2-hexadecanediol, (c) 1,2-hexanediol, (d) Tween 85 and (e) poly(vinylpyrrolidone) (PVP). Reproduced from reference [128] with permission from Elsevier.

Other specific methods have also been developed in order to increase the basal plane spacing. Physical adsorption of organic molecules on the surface of the modified montmorillonites can be carried out in order to fully organophilize the montmorillonite by eliminating the residual electrostatic forces of interaction. The area available per cation on the surface of the montmorillonite is generally larger than the area per cation of the conventional ammonium ions therefore leaving gaps in between on the surface after surface modification which keeps the surface partially polar. By physical adsorption of the organic molecules in these spaces, the gaps are completely filled and the platelets get more susceptible to the exfoliation in the polymers during compounding owing to the reduced forces. Figure 26A shows the process of physical adsorption and the various organic molecules adsorbed on the surface of the montmorillonite surface have been shown in Figure 26B [128]. These molecules (e.g. long alkyl chain alcohols) can adsorb physically in between the gaps generated after modification with ammonium ions by forming H-bonds with the OH groups present either in the inside structure of clay crystals or on the edges of the platelets. The adsorption has also been reported to take place on the preadsorbed water molecules in the clay interlayers [129,130,131,132]. Apart from that, bifunctional or multifunctional molecules can also be employed in order to generate much stronger adsorption on the filler surface. It was reported by the authors that there was an optimum amount of the adsorbent which could be adsorbed on the surface, after a critical amount, no further adsorption took place probably indicating the complete coverage of the surface. 

**Table 1:** Basal plane spacing values of various surface modified montmorillonites and vermiculites. Reproduced from reference [127].

Modification	Substrate, CECμ.eq/g^-1^	Basal spacing, nm
octadecyltrimethylammonium	montmorillonite, 880	1.84
octadecyltrimethylammonium	montmorillonite, 680	1.82
octadecyltrimethylammonium	montmorillonite, 900	1.85
octadecyltrimethylammonium	montmorillonite, 1000	2.14
dioctadecyldimethylammonium	montmorillonite, 880	2.51
dioctadecyldimethylammonium	montmorillonite, 680	2.45
trioctadecylmethylammonium	montmorillonite, 880	3.42
trioctadecylmethylammonium	montmorillonite, 680	3.29
benzylhexadecyldimethylammonium	montmorillonite, 880	1.88
benzylhexadecyldimethylammonium	montmorillonite, 680	1.85
docosyltriethylammonium	montmorillonite, 880	1.93
decylmethyloctadecylimidazolium	montmorillonite, 880	2.24
didocyldimethylammonium/dioctadecyldimethylammonium	montmorillonite, 880	2.28
didocyldimethylammonium/dioctadecyldimethylammonium	montmorillonite, 680	2.27
benzylhydroxyethylmethyloctadecyl ammonium	montmorillonite, 880	2.06
benzldibutylhydroxyethylammonium	montmorillonite, 880	1.52
benzyldi(hydroxyethyl)butyl ammonium	montmorillonite, 880	1.50
benzyltriethanolammonium	montmorillonite, 880	1.52
benzylhydroxyethylmethyloctadecyl ammonium	vermiculite, 1400	3.40
benzylhexadecyldimethylammonium	vermiculite, 1400	3.25

It was also reported that when long chain polymers were adsorbed on the surface, not only the organophilization of the surface was achieved, the thermal stability of the modified filler was also improved. These specifically modified fillers represent high potential fillers for complete exfoliation in the polymer matrices especially polyolefins when compounded with them at high temperatures.

Polymerization reactions on the surface of the clay have been earlier described. In the similar fashion [133], esterification reactions at the surface of the modified montmorillonite have also been reported to increase the filler basal plane spacing. The montmorillonite is first modified with a reactive modification e.g. containing OH groups which can then be subsequently reacted with the long chain carboxylic acid. 

**Figure 27:**
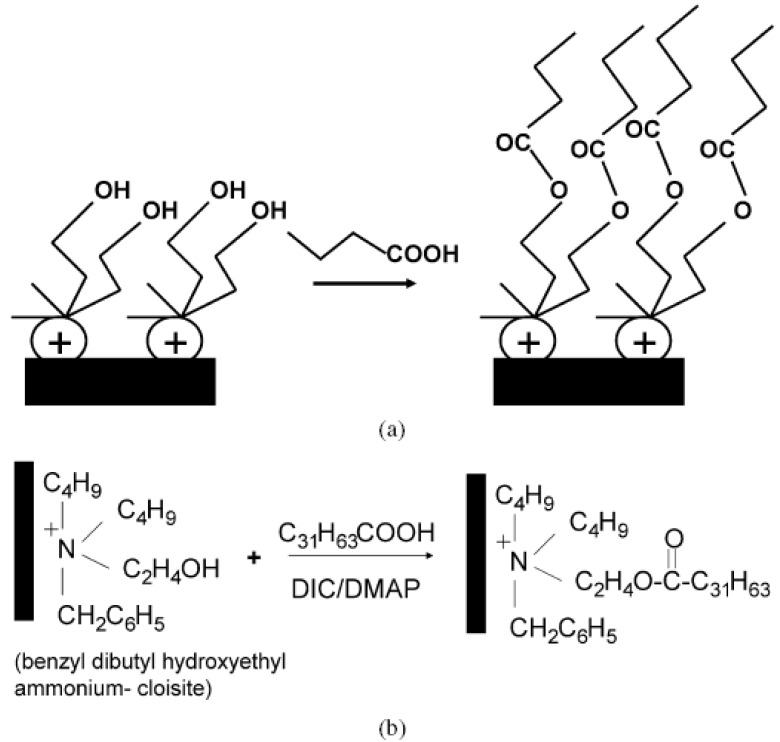
(a) Schematic of surface esterification reactions and (b) representation of the reaction with montmorillonite modified with benzyl dibutyl hydroxyethyl ammonium. Reproduced from reference [133] with permission from Elsevier.

The process is demonstrated in Figure 27. It was reported that the number of hydroxyl groups present in the surface modification significantly affected the extent of surface reaction. The presence of an octadecyl chain in the surface modification was also helpful in achieving higher basal plane spacing in the modified montmorillonite which subsequently helped to achieve higher extent of surface esterification reaction. By allowing more space for the reaction to proceed owing to the better swelling of the montmorillonite, high density brushes of long alkyl chains could be achieved which were successful in distancing the clay platelets significantly. The extent of surface reaction enhanced with increasing the amount of excess acid used for the reaction which subsequently increased the basal plane spacing. The re-reaction on the already reacted clay was also observed to improve the surface esterification. The advantage of this technology was also the achievement of long polymer chains chemically bound to the surface of layered silicates.

## 5. Nanocomposite Properties

### 5.1. Mechanical Properties

As mentioned earlier that it is the exfoliated platelets which contribute most to the improvement of nanocomposite properties. The increase in the aspect ratio of the filler due to the intercalation of large amount of polymer in the silicate interlayers enhances the organic-inorganic interfacial contacts thus leading to better stress transfer in the material. However, in reality, it is difficult to achieve the complete exfoliation of the silicate platelets and the platelets with varying thicknesses (i.e. stacks with varying number of platelets) are present in the composite materials. Therefore, it is important to relate this characteristic of the composite materials with the resulting composite properties. In order to account for the incomplete exfoliation of the filler, the following expression was suggested [134]:

$$t_{\text{particle}}=d_{001}^{*}(n-1)+t_{\text{platelet}}$$

**Figure 28:**
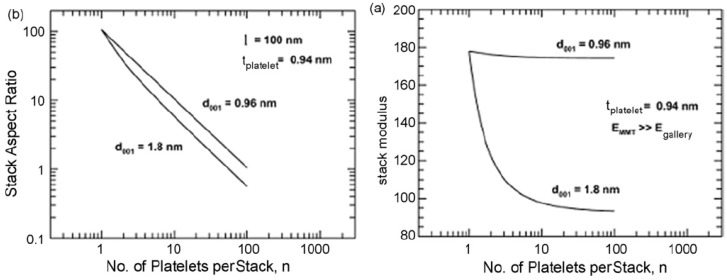
Stack aspect ratio as well as stack modulus as a function of the number of platelets in the stack. Reproduced from reference [134] with permission from Elsevier.

Stack aspect ratio as well as stack modulus as a function of the number of platelets in the stack was also described as shown in Figure 28. Increasing the number of platelets in the stack decreases the aspect ratio as well as the modulus of the material thus emphasizing the significance of the exfoliated platelets in property enhancement as well as need to achieve the platelet exfoliation for better composite properties at lower filler volume fractions.

Table 2 demonstrates the tensile strength of the various polymer nanocomposites reported in the literature [4]. The tensile modulus was reported to improve owing to the addition of the layered silicates. For example, the modulus increased by almost two-fold in the case of polyamide nanocomposites on addition of 5.3 wt% of modified clay. Similarly, other polymer matrices also exhibited increase in the modulus on the addition of small amount of filler. Improvement of the modulus in the case of polyolefin nanocomposites was not as high as the other polymers like polyamides or other polar polymers owing to the difficulties associated with the intercalation of non-polar chains inside the polar silicate interlayers thus requiring the use of compatibilizers.

**Table 2:** Young modulus of polymer nanocomposites. Reproduced from reference [4] with permission from Elsevier.

Nanocomposite	Clay Content (wt.%)	Young modulus (GPa)
PA6/MMT (*in situ* polymerization)	0	1.11
	4.7	1.87
	5.3	2.04
PA6(LMW)/MMT (melt intercalation)	0	2.82
	3.2	3.65
	6.4	4.92
PA6(MMW)/MMT (melt intercalation)	0	2.71
	3.1	3.66
	7.1	5.61
PA6(HMW)/MMT (melt intercalation)	0	2.75
	3.2	3.92
	7.2	5.70
PP(7.2% PP-g-MA)/OMLS	0	0.714
	7.2	0.838
PP(21.6% PP-g-MA)/OMLS	0	0.760
	7.2	1.010
EVA/Cloisite Na	0	0.0122
	3	0.0135
EVA/Cloisite 20A	0	0.0122
	3	0.0249
EVA/Cloisite 25A	0	0.0122
	3	0.0220
EVA/Cloisite 30B	0	0.0122
	3	0.0228
EVA/Nanofil 757	0	0.0122
	3	0.0116
EVA/Nanofil 15	0	0.0122
	3	0.0240
EVA/Somasif ME100	0	0.0122
	3	0.0124
EVA/Somasif MAE	0	0.0122
	3	0.021
Soft PU/30B (solution intercalation)	0	0.0075
	3	0.0138
	7	0.024
Soft PU/30B (melt intercalation)	0	0.0072
	3	0.0114
	7	0.0193
Hard PU/30B (solution intercalation)	0	0.050
	3	0.086
	7	0.134
Hard PU/30B (melt intercalation)	0	0.061
	3	0.081
	7	0.119
HDPE/o-MMT	0	1.020
	0.9	1.060
	1.8	1.250
	2.8	1.380
	4.8	1.360

**Figure 29:**
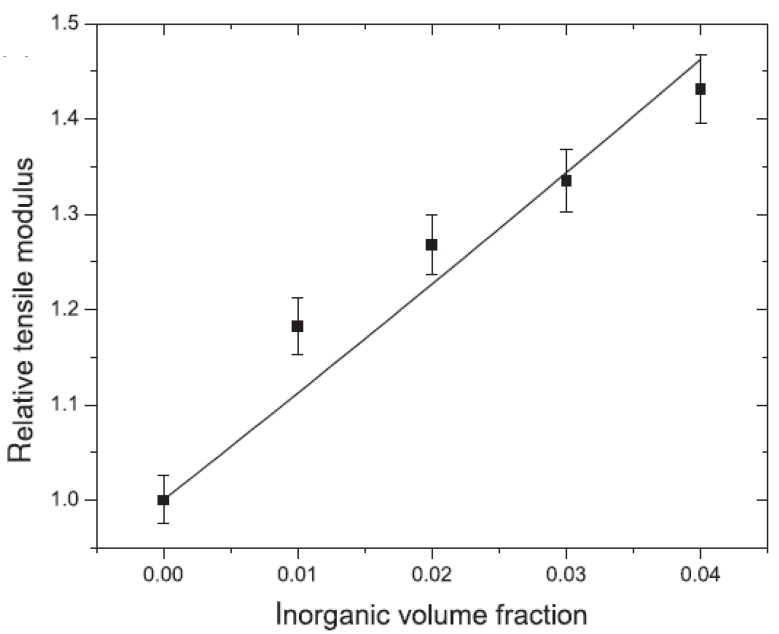
Improvement of the tensile modulus of the polypropylene nanocomposites as a function of the filler volume fraction. Reproduced from reference [43].

The tensile modulus is generally reported to increase as a function of filler volume fraction. Figure 29 demonstrates such an improvement in the tensile modulus of the polypropylene nanocomposites as a function of the amount of the layered silicate modified with dioctadecyldimethylammonium ions [43]. 

**Figure 30:**
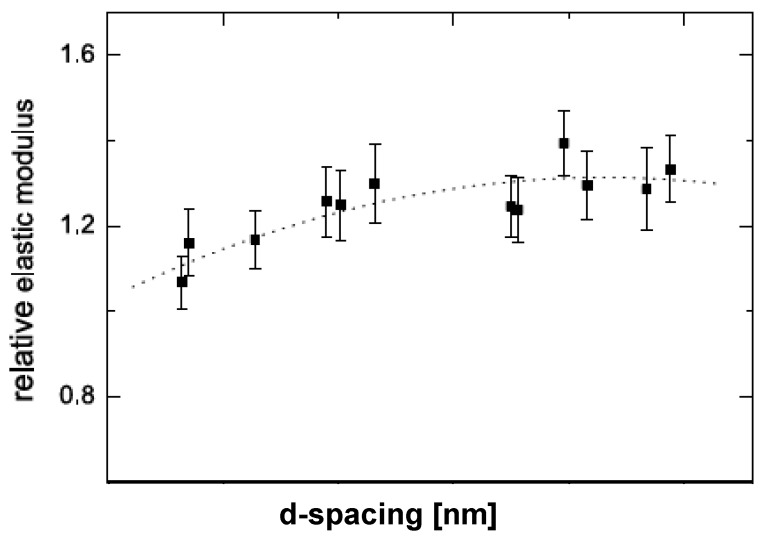
Increase in the elastic modulus of the composites as a function of the basal plane spacing of the filler. Reproduced from reference [135] with permission of Elsevier.

As is visible, the modulus increased linearly as a function of the filler fraction exhibiting an increase of 45% at 4 vol% filler as compared to the pure polypropylene. It has to be noted that the results in Figure 29 were achieved without the use of conventionally added compatibilizers. Osman *et al.* also reported similar nanocomposites with polyethylene without the use of compatibilizers [135]. The basal plane spacing of the filler was reported to be influencing the susceptibility of the filler to exfoliate. If the filler was modified with the ammonium ions with higher chain density in the molecules (like trioctadecylmethylammonium or tetraoctadecylammonium), the platelets were pushed further far apart leading to reduction in the electrostatic forces between them which helps to exfoliate the platelets in the polymer matrices during compounding. Figure 30 describes this notion. The fillers with higher basal plane spacing also led to increased elastic modulus in the composites owing to the increased extent of filler exfoliation. Thus, in such a case, there are no attractive forces between the polymer and the silicate which help to intercalate the polymer chains and the process is more kinetic in nature where the loosely held filler stacks are delaminated by the action of shear, thus kinetically trapping the platelets in the polymer matrix. Such a behavior is totally different from the case of polar polymers where the polar interactions between the filler surface and polymer chains lead to interaction of the polymer chains in the interlayers.

**Figure 31:**
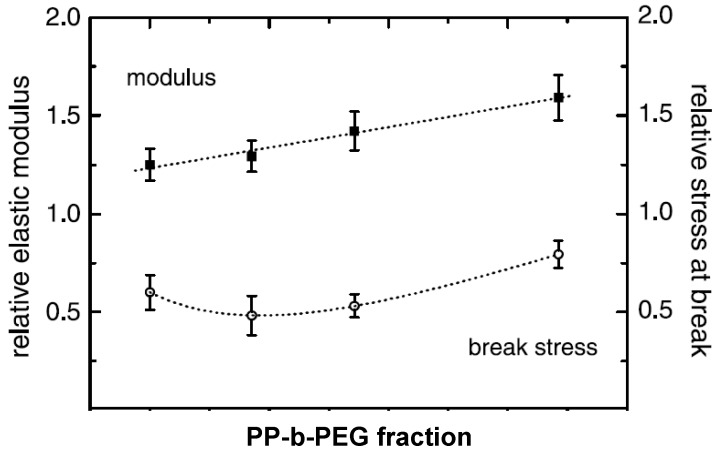
Increase in the tensile modulus of the polyethylene nanocomposites as a function of the amount of the compatibilizer. The filler content was fixed at 3 vol%. Reproduced from reference [136] with permission from Elsevier.

**Figure 32:**
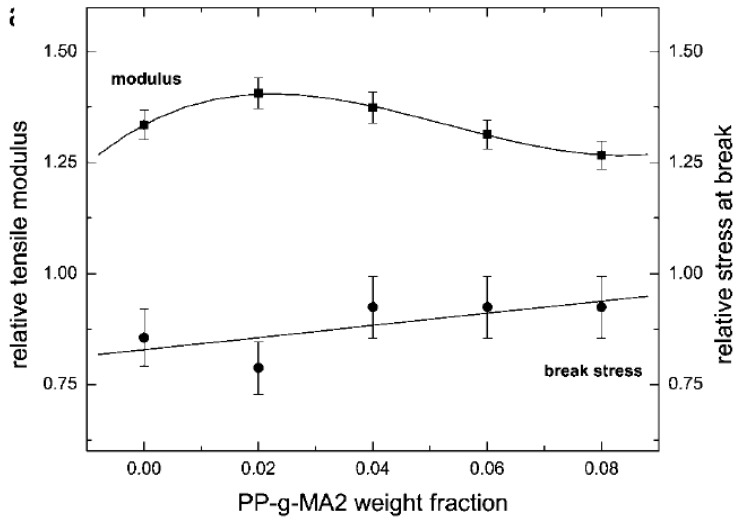
Tensile modulus and stress at break as a function of the amount of compatibilizer for the synthesis of polypropylene nanocomposites. Reproduced from reference [137].

Osman *et al.* [136] also reported preparation of polyethylene nanocomposites by using compatibilizers. The filler was modified with dioctadecyldimethylammonium and a block copolymer was used as compatibilizer. The block copolymer was reported to be better than other kinds of compatibilizers due to its well defined polar and non-polar blocks. The increasing amount of compatibilizer increased the extent of delamination of the filler leading to the generation of more exfoliated morphology in the composites which resulted in better tensile modulus as shown in Figure 31. However, in another study, it was reported that the tensile modulus of the composites increases only till a critical value of the compatibilizer [137]. If the compatibilizer is present in higher amounts than the critical value, then the tensile modulus is negatively impacted as shown in Figure 32 for polypropylene nanocomposites. Montmorillonite modified with dioctadecyldimethylammonium ions was used and low molecular weight PP-g-MA (MA content 4%) was used as a compatibilizer. 

It was reported that the mechanical properties of the composites are jointly affected by plasticization and exfoliation. At low weight fraction of compatibilizer, the exfoliation effect is more significant, thus leading to increased tensile modulus. However, the effect of exfoliation becomes less pronounced at the higher amounts of compatibilizer as the low molecular weight compatibilizer then plasticizes the matrix reducing the modulus even though the extent of delamination of the silicate is increased. 

**Figure 33:**
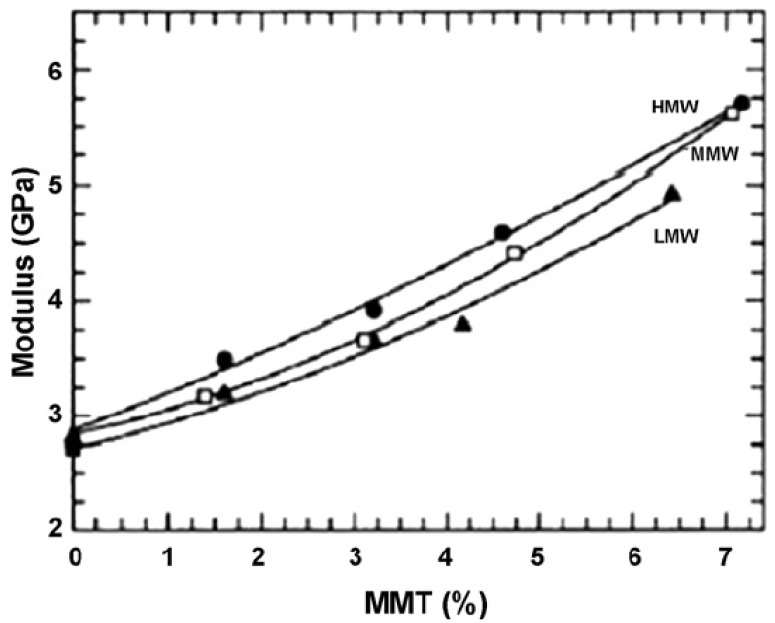
Increase in the tensile modulus as a function of the amount of montmorillonite fraction. Three matrices with low, medium and high molecular weights are depicted. Reproduced from reference [93] with permission from Elsevier.

**Figure 34:**
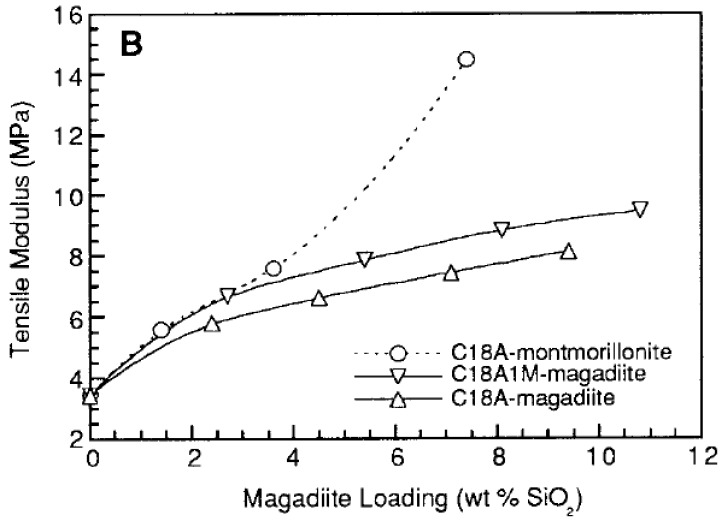
Tensile modulus of the epoxy nanocomposites as a function of filler volume fraction. Reproduced from reference 139 with permission from American Chemical Society.

Polyamide nanocomposites with low, medium and high molecular weights were employed for the synthesis of nanocomposites [93,138]. It was observed that the stiffness increases with increasing molecular weight of the matrix even though the modulus was the same for the neat polyamide matrices. This behavior is demonstrated in Figure 33. Similarly, epoxy nanocomposites with different inorganic materials have also been reported. Figure 34 reports the tensile modulus of the composites as a function of the amount of magadiite [139].

The magadiite was either modified with octadecyl-ammonium or methyloctadecylammonium. For comparison, the octadecylammonium montmorillonite system was also studied. A much significant increase of the tensile modulus for the montmorillonite based nanocomposites for filler contents higher than 4 wt% was observed. The authors suggested that behavior is caused by the difference in layer charge density for magadiite and montmorillonite. Organo-magadiites have a higher layer charge density and consequently a higher alkylammonium content than organo-montmorillonite. As the alkylammonium ions interact with the epoxy resin while polymerizing, dangling chains are formed. More of these chains are thus formed in presence of organo-magadiites. These dangling chains are known to weaken the polymer matrix by reducing the degree of network cross-linking, then compromising the reinforcement effect of the silicate layer exfoliation. The behavior of tensile strength of nanocomposites is not as clear as the tensile modulus and the tensile strength is more of a function of the generated morphology. Therefore, in many reported studies, the strength was observed to increase on the incorporation of clay, whereas many other reports, a decrease in the tensile strength as a function of filler fraction. Table 3 reports the tensile strength values for many polymer nanocomposite systems [4]. 

**Table 3:** Tensile strength values of various polymer nanocomposite systems. Reproduced from reference [4] with permission from Elsevier.

Nanocomposite	Clay Content (wt.%)	Tensile Strength (MPa)
PA6/MMT (*in situ* polymerization)	0	68.6
	4.7	97.2
	5.3	97.3
PA6(LMW)/MMT (melt intercalation)	0	69.2
	3.2	78.9
	6.4	83.6
PA6(MMW)/MMT (melt intercalation)	0	70.2
	3.1	86.6
	7.1	95.2
PA6(HMW)/MMT (melt intercalation)	0	69.7
	3.2	84.9
	7.2	97.6
PMMA/OMLS	0	53.9
	12.6	62.0
PS/OMLS	0	28.7
	17.2	23.4
	24.6	16.6
EVA	0	28.4
EVA/Cloisite Na	3	25.9
EVA/Cloisite 20A	3	25.8
EVA/Cloisite 25A	3	26.2
EVA/Cloisite 30B	3	30.7
EVA/Nanofil 757	3	27.6
EVA/Nanofil 15	3	26.7
EVA/Somasif ME100	3	24.5
EVA/Somasif MAE	3	25.1
Soft PU/30B (solution intercalation)	0	45
	3	31
	7	21
Hard PU/30B (solution intercalation)	0	58
	3	44
	7	34
PU/MMT	0	5.9
	5	6.2
	10	6.5
	21.5	8.3
PE/JS	0	22
	5	25
	10	27
	15	28
PE/DM	0	22
	5	21
	10	23
	15	24
HDPE/o-MMT	0	27
	0.9	26
	1.8	26
	2.8	26
	4.0	25

**Figure 35:**
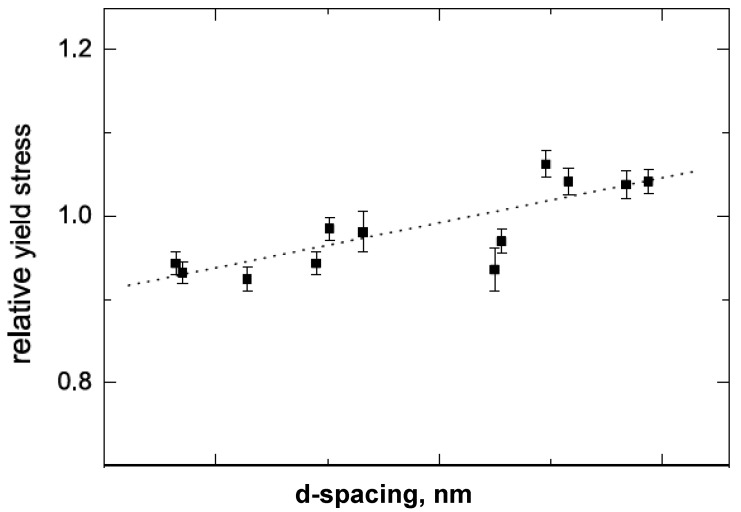
Relative yield stress as a function of the filler basal plane spacing used for the synthesis of polyethylene nanocomposites. Reproduced from reference [135] with permission from Elsevier.

**Figure 36:**
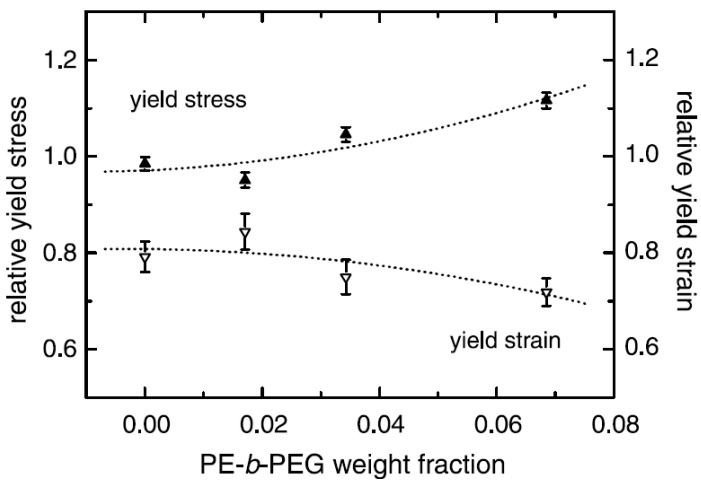
Yield stress and yield strain of the polyethylene nanocomposites as a function of the amount of the block copolymer based compatibilizer. Reproduced from reference [136] with permission from Elsevier.

The tensile strength was also reported to be a function of the basal plane spacing of the filler. Osman *et al.* [135] reported by using a large number of filler surface modification that similar to tensile modulus of the composites, tensile strength of the composites had also the tendency to increase as a function of increasing basal plane spacing in the filler owing to the increased extent of exfoliation for the filler where the interlayer spacing is higher. The behavior is represented in Figure 35. The tensile strength of polyolefin nanocomposites as function of amount of compatibilizer in the system is also reported in Figure 36 [136]. The increasing amount of compatibilizer leads to improvement in the tensile strength due to increase extent of filler delamination. 

In an interesting study for the generation of PET nanocomposites, in-situ intercalative polymerization was used followed by extrusion through the die of a capillary rheometer [140]. The extrudates were stretched through the die of the rheometer into various draw ratios. When the organoclay was increased from 0 to 3 wt% in hybrids at draw ratio, DR= 1, the strength linearly improved from 46 to 71 MPa, and the modulus from 2.21 to 4.10 GPa. On the other hand, it is quite interesting to note the effect of DR on the tensile strength and modulus of PET and PET nanocomposite fibers. For pure PET, the strength and modulus increased from 46 to 51 MPa and 2.21 to 2.39 GPa, respectively, as the DR was increased from 1 to 16.

Stress at break values for the polymer nanocomposites based on nylon [141] and rubber [142] generally show an increase in value as compared to the pure polymer. This increase is explained on the basis of the polar or ionic interactions between the polymer chains and the polar silicate surface. The increase in the stress at break is more pronounced in polyamide nanocomposites owing to higher extents of exfoliation as well as ionic bonds with the silicate surface [39]. However, in the case of non-polar polymers, as there are no attractive interactions between the polymer chains and the silicate surface, therefore, the break stress generally is observed to decrease or increases only slightly in these polymer nanocomposites. Incorporation of compatibilizer in the system, however, increases the polarity of the system and increases the intercalation of the polymer in silicate interlayers owing to the interfacial interactions between the compatibilizer chains and the silicate surface. As a result, the break stress increases in the compatibilized polyolefin nanocomposite systems as shown in Figure 31 and Figure 32. The break stress was also observed to increase as a function of the amount of compatibilizer in the system. Elongation at break of polymer nanocomposites has also been reported to be more dependant on the morphology of the nanocomposites. Therefore, studies reporting both an increase and decrease of the elongation at break in the nanocomposites have been reported [143,144,145]. 

Impact properties of the composites have also been contradictory in the reported studies. Impact strength of the nylon composites was reported to decrease as function of the concentration of the filler [146]. Other studies also reported little or no change in the impact strength of the composites [147]. Other authors also reported an increase in the impact strength as reported earlier. Oya reported an increase in the notched izod impact strength from 2 to 3.4 kJ·m^-2^ for 3 wt% polypropylene nanocomposites. Montmorillonites were modified with octadecylammonium and PP-g-MA was used as compatibilizer [114,119]. Similarly, Zhang *et al.* also reported that the impact strength increase from 9.4 kJ·m^-2^ for the pristine PP to 25.9 kJ·m^-2^ for the PP composites compatibilized with PP-g-MA. In these composites, dioctadecyldimethylammonium ions were exchanged on the silicate surface. Surprisingly the above mentioned increase was achieved at a filler loading of only 0.1 wt% [113]. 

**Figure 37:**
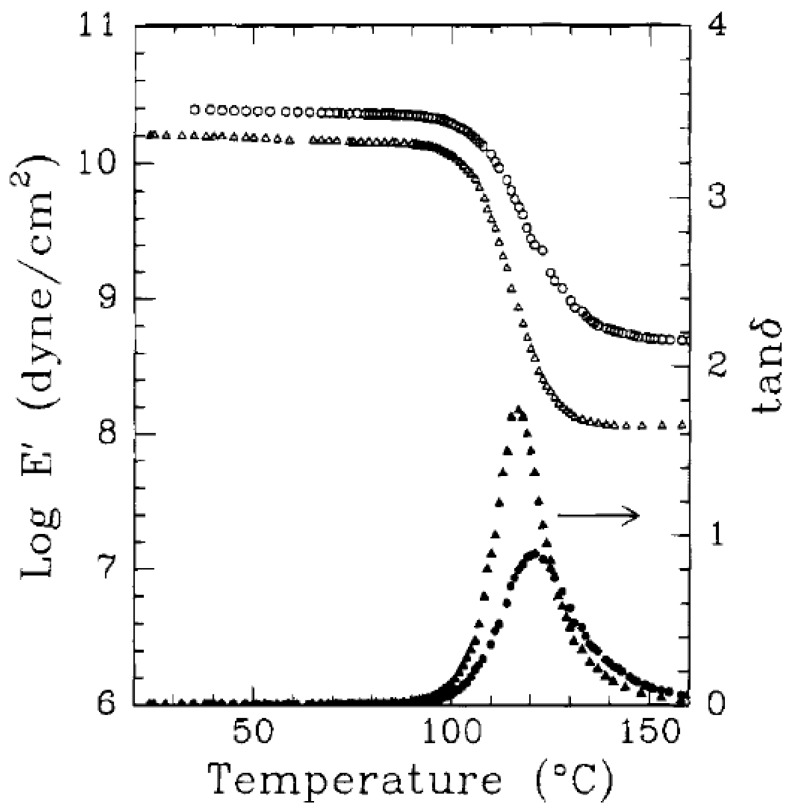
Temperature dependence of E’ and tan δ for fully cured DGEBA/BDMA and OMTS/DGEBA/BDMA. Reproduced from reference [86] with permission from The American Chemical Society.

**Figure 38:**
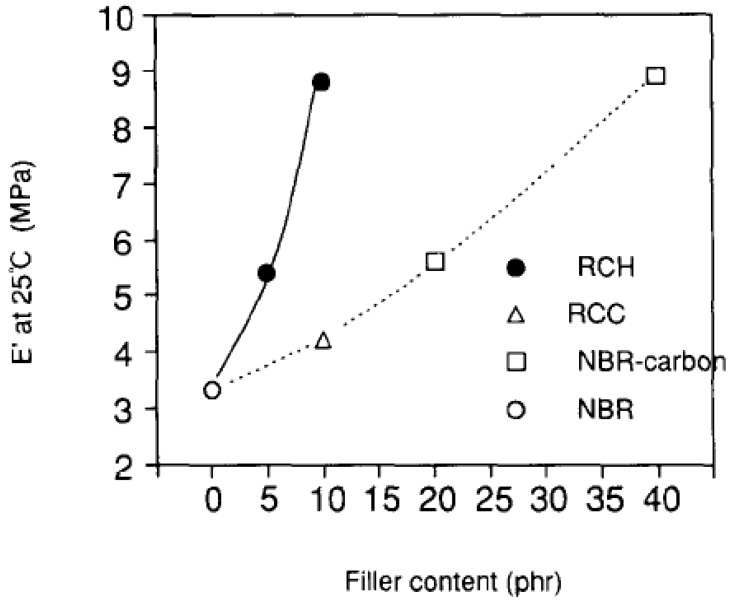
Elastic modulus of rubber clay hybrids as a function of filler volume fraction. Reproduced from reference [70] with permission from Elsevier.

Dynamic mechanical analysis (DMA) has also been extensively used for the polymer nanocomposites to measure the response of a given material to a cyclic deformation as a function of the temperature. As a result, the storage modulus (E’), corresponding to the elastic response to the deformation, the loss modulus (E’’), corresponding to the plastic response to the deformation and tan δ, which is the ratio of the elastic to loss modulus have been reported. In one such study [86], as shown in Figure 37, the temperature dependence of the tensile storage modulus, E’, and tan δ of the epoxy system with and without the organically modified montmorillonite are reported. The shift and broadening of the tan δ peak to higher temperatures showed an increase in nanocomposite T_g_ and broadening of the glass transition. Broadening and increase of T_g_ have been reported for other nanocomposites too and is generally attributed to restricted segmental motions near the organic-inorganic interface. It has also been observed from the other studies that the storage modulus increases upon the addition of the filler and this increase is more significant above the glass transition temperature. In Figure 37 too, below T_g_, E’ in the glassy region below T_g_ is approximately 58% higher in the nanocomposite compared to the pure epoxy. However, the nanocomposite exhibits a plateau modulus approximately 4.5 times higher than the unmodified epoxy in the rubbery region. Similarly, the rubber clay hybrid (RCH) [70] was also reported to have much higher elastic modulus than the carbon reinforced or conventional composites as shown in Figure 38. Other polymer systems have also been analyzed similarly for the dynamic mechanical properties [148,149,150,151].

### 5.2. Barrier Properties

Barrier properties of the nanocomposites are of immense importance for a number of applications. It is also generally assumed that the improvement in the mechanical properties of the composites would automatically lead to enhancement in the barrier properties, which is not always true. Barrier properties are more strongly affected by the changes at the organic inorganic interface, therefore, separate studies are required to completely understand the barrier performance of the systems. 

**Figure 39:**
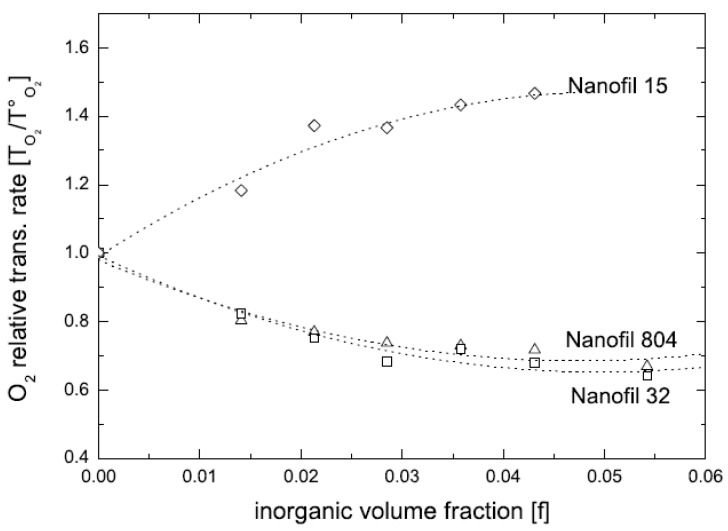
Oxygen permeation through the polyurethane nanocomposites as a function of filler volume fraction. Reproduced from reference [87] with permission from The American Chemical Society.

Barrier properties have not been studied as much as the mechanical performance. Messersmith and Giannelis [20] reported that the permeability of water through the PCL nanocomposites was dramatically reduced. Xu *et al.* reported that the relative water vapor permeation (P_c_/P_0_) of a poly(urethane urea)-Cloisite 15A (Southern Clay, dimethyl dihydrogenated tallow ammonium) composite decreased 5-fold at a 6 vol% filler concentration [152]. Chang *et al.* observed that the oxygen permeation through polyurethane decreased by 50% or 15% at 4 wt% of montmorillonite surface treated with hexadecylammonium or dodecyltrimethylammonium salts, respectively, and by 35% at 4 wt% Cloisite 25A (Southern Clay, 2-ethylhexyldimethyl hydrogenated tallow ammonium) [153]. However the data given in this reference were not normalized to the film thickness, which varied between 10 and 15 μm, so that those numbers could be misleading. Tortora *et al.* claimed that the water vapor permeation through a polyurethane composite showed a "remarkable decrease" up to 20 wt% organoclay concentration [154]. An interesting study was reported for polyurethane nanocomposites by Osman *et al.* [87]. Montmorillonites modified with different surface modifications shown in Figure 22 were chosen and their interactions with the polyurethane matrix were studied. The oxygen permeation through the composite film has been described in Figure 39 as a function of filler volume fraction. The oxygen permeation in the composites containing montmorillonites modified with modifications having hydroxyl or benzyl groups in the structure decreased as a function of filler volume fraction. However, the permeation increased in the composites which contained montmorillonite modified with dioctadecyldimethylammonium. It was suspected that the incompatibility of the polyurethane matrix with the non-polar surface modification led to the generation of micro voids at the interface which cause the increase in the oxygen permeation and as a result, the oxygen permeation increases on increasing the filler in the system. Water vapor permeation was, however, not affected due to fundamental differences in the principles of diffusion of the two permeants. 

**Figure 40:**
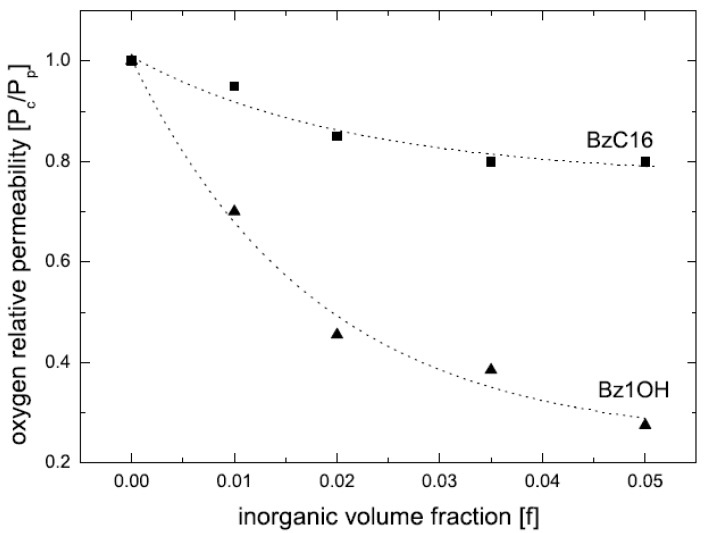
Oxygen permeation of the epoxy nanocomposites as a function of filler volume fraction. Reproduced from reference [45] with permission from The American Chemical Society.

The authors also showed similar behavior in epoxy nanocomposites, where a number of surface modifications as shown in Figure 12 were reported [45]. It was also observed that the initial basal plane spacing of the filler was not necessary to be high to achieve exfoliation of the filler. This is shown in Figure 40 where the oxygen permeation of epoxy nanocomposites containing montmorillonites modified with bezylhexadecyldimethylammonium (BzC16) and benzyldibutyl(2-hydroxyethyl)-ammonium (Bz1OH) have been plotted as function of filler volume fraction. The Bz1OH modified filler though had a low basal plane spacing of 1.52 nm, however, it led to the synthesis of largely exfoliated nanocomposites owing to the better match of polarity between the polar epoxy polymer and the surface modification. The BzC16 filler on the other hand, though had a larger initial basal plane spacing owing to the hexadecyl chains in the molecules, however the generated composite morphology is more intercalated owing to the mismatch of polarity between the surface modification molecules and polymer chains. 

The oxygen permeation through the composite films also shows corresponding behavior with Bz1OH modified filler system leading to a much higher reduction in the oxygen permeation. Table 4 also lists the oxygen and water vapor permeation trough the nanocomposite films containing 3 vol% of the fillers modified with different surface modifications (see Figure 12 for details of the modifications).

**Table 4:** Oxygen and water vapor permeation though the epoxy nanocomposites at 3 vol% filler fraction. Reproduced from reference [45] with permission from The American Chemical Society.

Composite	Oxygen permeability coefficient [cm^3^.μm/(m^2^.d.mmHg)]	Water vapor trans. Rate [g.μm/(m^2^.d.mmHg)]
Neat Epoxy	2.0	10
Cloisite Na^+^	1.6	23
Bz1OH	0.77	6.7
Bz2OH	0.78	5.8
Bz3OH	1.0	7.1
BzC18OH	2.2	5.7
BzC16	1.6	5.3
2C18	3.7	6.8

**Figure 41:**
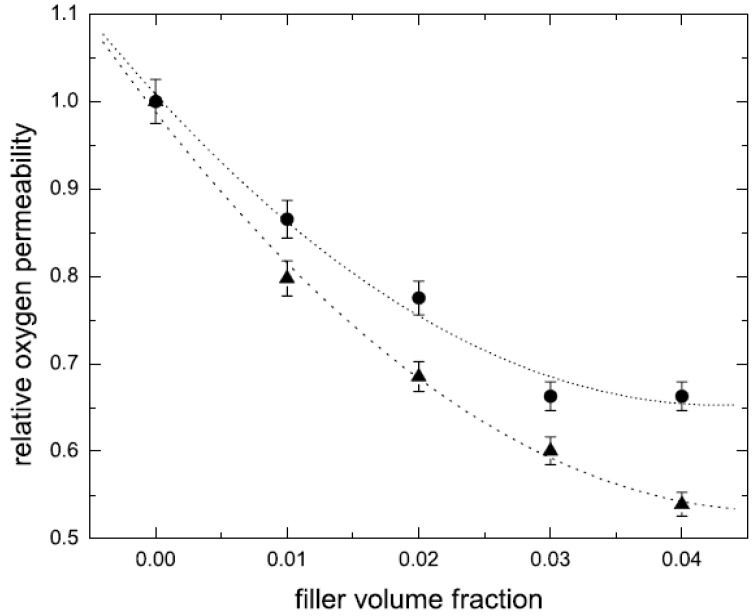
Oxygen barrier properties of polypropylene nanocomposites as a function of filler volume fraction. The montmorillonites have been modified with ammonium based (●) and imidazolium based (▲) modifications. Reproduced from reference 50 with permission from Elsevier.

Oxygen barrier properties of the polyolefin composites are also of interest owing to their poor oxygen resistance. Polyolefins are very resistant to water or water vapor, thus generating oxygen barrier functionality in such materials would make these materials versatile for barrier applications. Gorrasi *et al.* reported the reduction in diffusion of organic vapors trough the composites on incorporation of clay, which was correlated to the filler volume fraction [155]. Manias reported that water vapor and oxygen barrier properties were reduced by half at 4 wt% filler loading in PP nanocomposites [156]. Barrier properties of polypropylene nanocomposites without the use of compatibilizer were also reported [49]. In these composites, utmost care was taken in order to wash off the filler surface any excess surface modification agent which may degrade during compounding at high temperature thus affecting the barrier properties. The compounding temperatures was also not too high so as to avoid the thermal degradation of the ammonium ions ionically bound to the surface as well as polymer, but the temperature was high enough to ensure homogenous mixing of the polymer with filler. Oxygen permeation results of the composites containing montmorillonite modified with dioctadecyldimethyl-ammonium are shown in Figure 41 as a function of filler volume fraction. The oxygen permeation decreased by 35% at 4 vol% of the filler in the composite. Recently, barrier properties of polypropylene nanocomposites with montmorillonites modified with more thermally stable imidazolium based surface modifications were also reported [50]. The thermally stable imidazolium based modifications are much more stable at the polymer compounding temperatures than the conventional ammonium based modification. The oxygen permeation properties of the polypropylene composites as shown in Figure 41 are also correspondingly improved. A reduction of almost 50% in the oxygen permeation was achieved at 4 vol% of the imidazolium modified montmorillonite. The authors stressed that by optimizing the filler surface modification, the improvement of the composite properties can be achieved without the use of conventionally used compatibilizers. Cation exchange capacity of the layered silicate as well as the chain density of the ammonium modifications were observed to improve the barrier resistance of the polymer nanocomposites similarly as observed for mechanical performance. 

**Figure 42:**
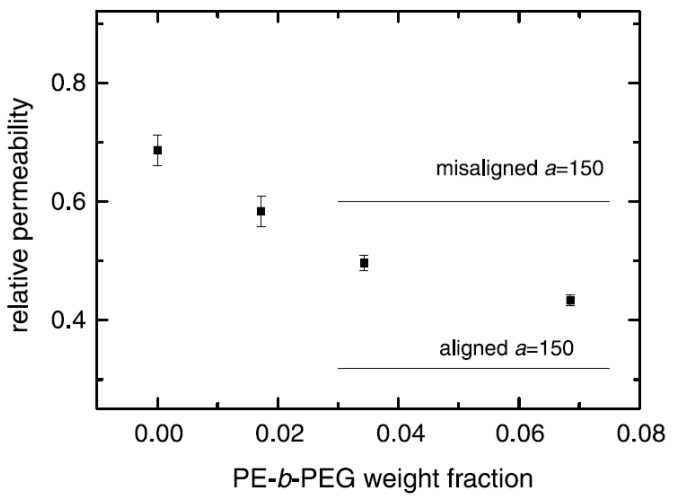
Relative oxygen permeability through the polyethylene nanocomposite films as function of block copolymer compatibilizer amount in the composite. Reproduced from reference [136] with permission from Elsevier.

Barrier properties of polyethylene nanocomposites with the use of block copolymer a compatibilizer have also been reported [136] as shown on Figure 42. The montmorillonite was modified with dioctadecyldimethylammonium and the filler volume fraction was fixed at 3 vol%. 

As shown in Figure 42, the oxygen permeation through the composite films decreased as a function of the amount of compatibilizer in the composite owing to the better delamination of the filler platelets. This increased extent of exfoliation then translates into significant reduction in the oxygen permeation through the composite films. However, as mentioned earlier, it is also possible that the increased amount of compatibilizer leads to extensive plasticization of the matrix thus counterbalancing the effect of exfoliation. In such instances, the barrier properties were observed to either remain unaffected or slightly deteriorate [137]. Similarly, other studies have also reported the permeation properties of the polymer layered silicate nanocomposites [157,158,159]. 

### 5.3. Thermal Stability and Flame Retardancy

As mentioned earlier, TGA is the most commonly used method for the determination of the thermal behavior of the modified fillers as well as polymer nanocomposites. Many authors have reported that the incorporation of the layered silicate in the polymer matrix leads to enhancement of the thermal stability of the polymer material. The layered silicates were postulated to act as superior insulators and mass transport barrier to the volatile products generated during decomposition. The silicate materials also assisted in the formation of char after thermal decomposition [160,161,162,163]. 

Bandyopadhyay *et al.* reported the improved thermal stability of the biodegradable nanocomposites consisting of poly(lactic acid) and organically modified fluorohectortie and montmorillonite [164]. It was observed by the authors that poly(lactic acid) intercalated in the fluorohectorite or montmorillonite interlayers resisted the thermal degradation. 

**Figure 43:**
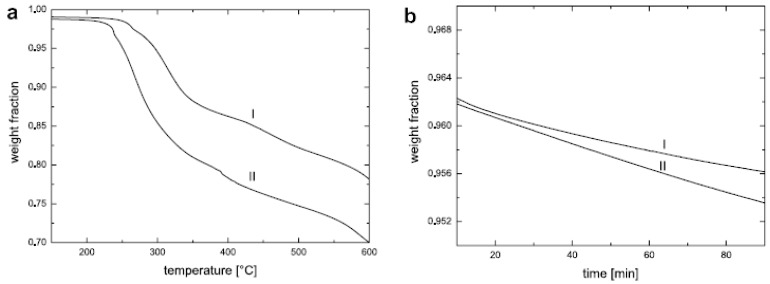
(a) TGA thermograms of the montmorillonite modified with 1-decyl-2-methyl-3-octadecylimidazolium (I) and dioctadecyldimethylammonium (II) and (b) dynamic thermogravimetric analysis of the modified montmorillonites. Reproduced from reference [50] with permission from Elsevier.

**Figure 44:**
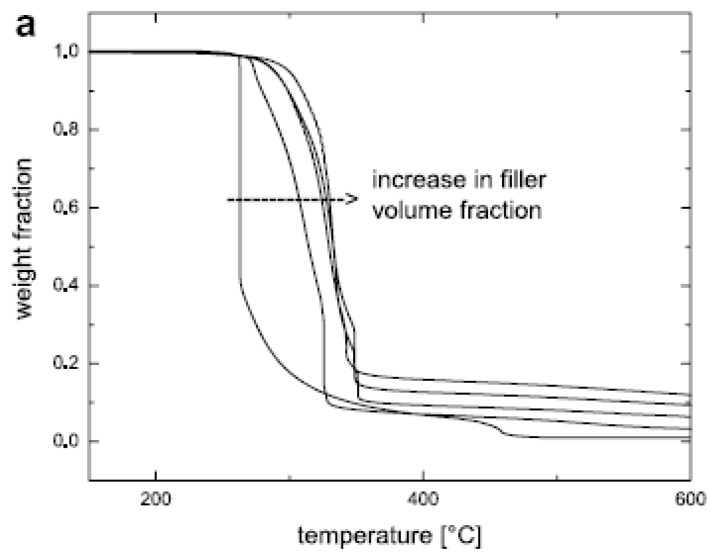
TGA thermograms of the polypropylene nanocomposites as a function of filler volume fraction. Reproduced from reference [50] with permission from Elsevier.

Paul *et al.* [165] also observed an increase in the thermal stability of the PLA nanocomposites as a function of the amount of clay in the composite. Maximum improvement in the thermal stability was observed at 5 wt% of the filler. Mittal also reported the thermogravimetric analysis of polypropylene nanocomposites with imidazolium modified montmorillonites [50]. The imidazolium surface modification was much more thermally stable than the conventionally used ammonium modifications as shown in Figure 43. The onset of degradation in the case of imidazolium based montmorillonite was roughly 50 °C higher than the ammonium counterpart. The peak degradation temperature was also significantly higher. The dynamic TGA under isothermal conditions was also performed on the montmorillonites with two modifications. Ammonium treated clay showed faster thermal degradation than the imidazolium counterpart. Although the difference in the amount of degradation of the two organo-montmorillonites is not significant, but owing to the fact that the main mechanism of degradation being the breakage of the weaker C–N bond, therefore, breaking of even small number of such bonds can seriously change the structure of the surfactant and its interaction with the polymer. The thermal behavior of the polypropylene nanocomposites with imidazolium modified montmorillonite is shown in Figure 44. Addition of only 1 vol% of the inorganic filler fraction improved the thermal behavior tremendously. Further additions also significantly improved the thermal degradation behavior of the composites with the composite containing 4 vol% of the filler showing the best thermal response. Thus, the thermal behavior of the polypropylene nanocomposites was synergistically improved on the incorporation of the clay. 

Thermal stability studies have also been carried out in mediums other than air [166,167,168]. Thermal decomposition of ethylene vinyl acetate (EVA) nanocomposites was investigated using helium and air [168]. In helium, EVA nanocomposites exhibit a negligible reduction in thermal stability compared to virgin EVA. However, when analyzed in air, the nanocomposites exhibit a large increase in thermal stability, as the maximum of the second degradation peak shifted by 40 °C to higher temperature, while the maximum of the first decomposition peak remained unchanged. The improved thermal stability was explained on the basis of the formation of protective char under oxidative conditions.

**Figure 45:**
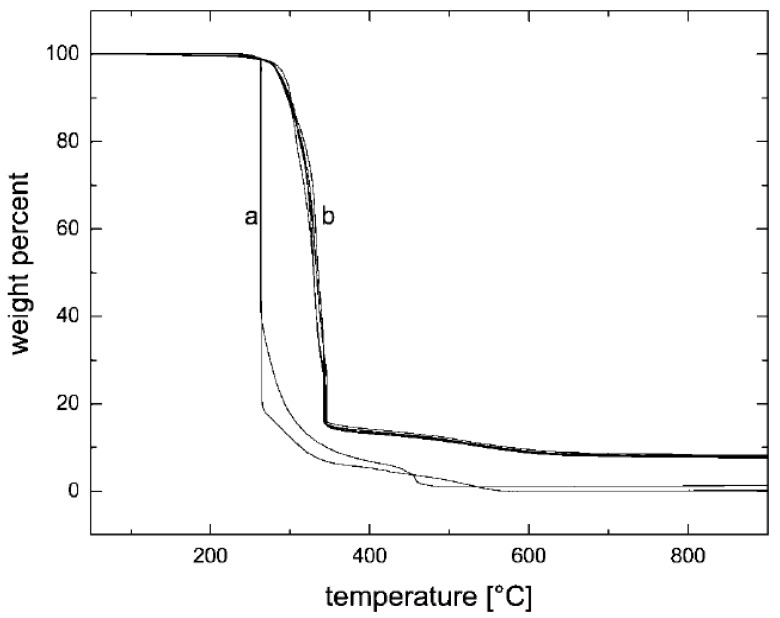
TGA thermograms of pure polymer and pure PP-g-MA compatibilizer (curves in group ‘a’) and polypropylene nanocomposites with 3 vol% of filler and containing 0, 2, 4, 6, 8 wt% of the compatibilizer (curves in group ‘b’). Reproduced from reference [137].

As low molecular weight compatibilizers are generally added for the synthesis of polyolefin nanocomposites, therefore, it is also of interest to analyze the thermal behavior of the these nanocomposites in order to ascertain the possible thermal degradation of compatibilizer chains during the compounding. Mittal reported the comparison of the TGA thermograms of the pure polypropylene and pure compatibilizer with the polypropylene nanocomposites containing 0, 2, 4, 6 and 8 wt% of compatibilizer as shown in Figure 45 [137]. All the composites showed similar thermal response irrespective of the amount of compatibilizer and the thermal stability of the composites was much better than the pure polymers. It was clear from the thermograms that irrespective of the low molecular weight of the compatibilizers, the presence of compatibilizer in the composites did not lead to any unwanted premature thermal degradation of the composites. Thus, the incorporation of organically modified filler improved the thermal behavior and the thermal response was not affected by the presence of low molecular weight compatibilizer in the composite.

**Figure 46:**
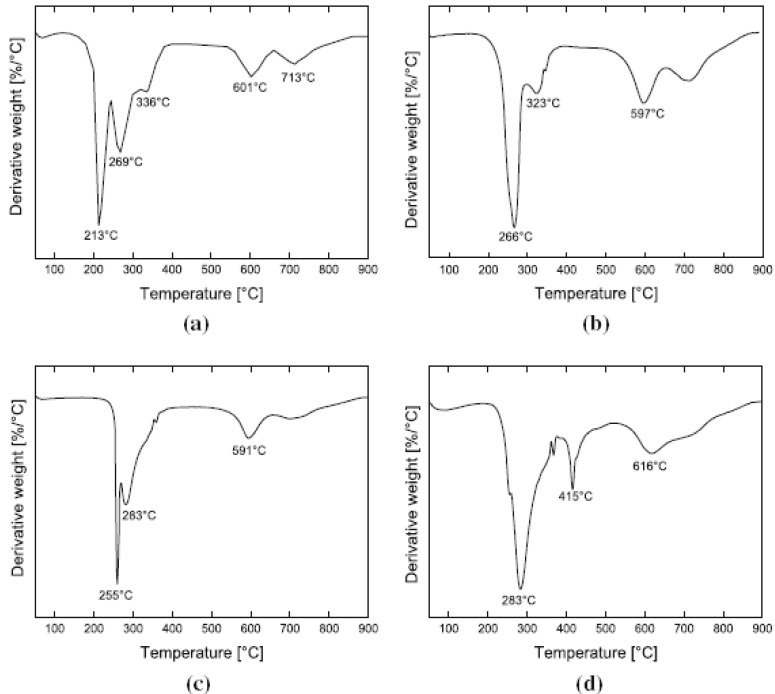
TGA thermograms of (a) commercially modified and (b) self prepared montmorillonite treated with benzylhexadecyldimethylammonium and (c) commercially modified and (d) self prepared montmorillonite treated with dioctadecyldimethyl-ammonium. Reproduced from reference [47].

The thermally stable modification on the filler surface has been mentioned earlier to improve the composite properties owing to improved thermal stability [50]. In recent study, the extent of cleanliness of the filler surface in the composite properties has also been underlined [47,169]. The commercially available surface modified montmorillonites are generally observed to contain an excess of the surface modification molecules in the interlayers present as pseudo bilayers unbound to the filler surface. These unbound surface modification molecules degrade at lower temperatures than the ionically bound molecules thus lowering the thermal stability of the modified montmorillonite. Figure 46 shows the TGA thermograms of commercially procured montmorillonites modified with benzylhexadecyldimethylammonium and dioctadecyldimethylammonium ions (a and c respectively). Figure 46b and d represent the TGA thermograms of the montmorillonites with the same ammonium ions but montmorillonites were self modified and rigorously washed after modification. 

Cone calorimetry is the widely used method for the analysis of the flame retardancy of the polymer nanocomposites. Parameters like heat release rate (HRR), peak of heat release rate (PHRR) and heat of combustion etc. are measured by this technique by exposing the samples to a defined heat flux. Reduction in the peak HRR is the most obvious signal of the flame retardant nature of the material. 

**Figure 47:**
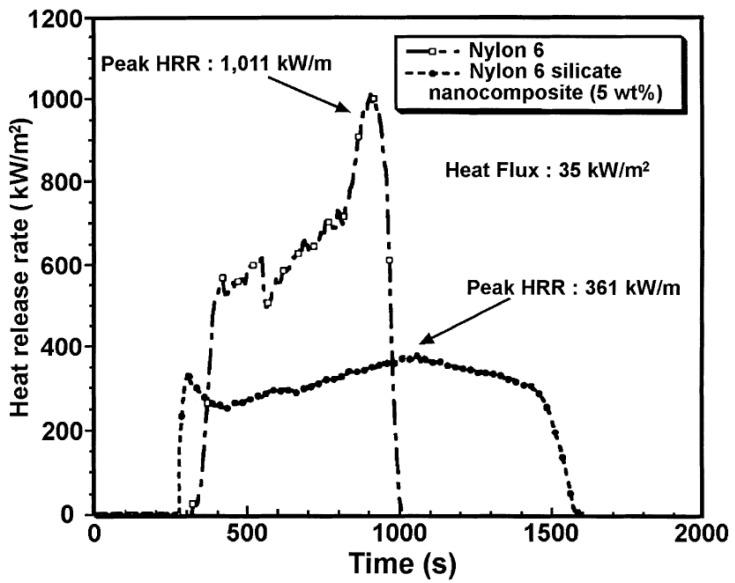
Comparison of the HRR for pure nylon 6 and nylon 6 nanocomposites containing 5 wt% of the filler. Reproduced from referebce [170] with permission from Elsevier.

Table 5 lists the cone calorimeter data of various polymer nanocomposites systems [4]. It is obvious from the values of HRR and PHRR that the incorporation the layered silicates in the polymers leads to improvements in the flame retardancy of the composite materials. The reduction in PHRR is generally a function of amount of clay in the composite, its aspect ratio and surface charge density. The reported studies have also suggested that the primary parameter responsible for the lower HRR in the case of nanocomposites is the mass loss rate (MLR) during combustion, which is significantly reduced in value compared to the pure polymer. Figure 47 represents the flame retardancy improvement in the nylon 6 nanocomposites on the incorporation of 5 wt% of clay [170]. A 63% reduction in the peak HRR was reported by the authors. 

**Table 5:** Cone calorimetric data of various polymer nanocomposite systems. Reproduced from reference [4] with permission from Elsevier.

Sample	PHRR(kW/m^2^)	HRR_ave_ (kW/m^2^)	SEA_ave_ (m^2^/Kg)
PA6	1010	603	197
PA6/2% OMLS	686	390	271
PA6/5% OMLS	378	304	296
PS	1120	703	1460
PS silicate mix3%	1080	715	1840
PS Nanocomposites 3%	567	444	1730
PSw/DBDPO/Sb_2_O_3_ 30%	491	318	2580
PS	1230		1315
PS/MAST-Hect 1%	1011		1336
PS/MAST-Hect 3%	894		1332
PS/MAST-Hect 5%	728		1327
PPgMA	1525	536	704
PPgMA nanocomposites 2%	450	322	1028
PPgMA nanocomposites 4%	381	275	968
EVA/Na+ 5%	1200		
EVA/Cloisite 30B 3%	860		
EVA/Cloisite 30B 5%	780		
EVA/Cloisite 30B 10%	630		
EVA	2303		430
EVA/30B	1174		670
EVA/30BHect	1289		593
EVA/30BMag	2010		476
EVA/MMT	1959		517
PU (I)	2561	741	176
PU (I)/o-MMT	918	344	305
PU (II)	2254	637	235
PU (II)/o-MMT	641	363	412
PU (III)	2647	768	165
PU (III)/o-MMT	848	444	172
PU (IV)	2664	775	235
PU (IV)/o-MMT	797	435	412
PE	1470	510	
PE/JS 2%	670	440	
PE/JS 5%	320	450	
PE/JS 10%	540	380	
PE/JS 15%	390	280	

Other reported studies on the flame retardancy properties of nanocomposites also confirm that only a small amount of filler is required to significantly improve the flame retardancy of the composites. Also, the silicates are more environmentally friendly materials than some flame retardants. The incorporation of the flame retardants also deteriorates the physical properties or color of the polymer materials, which can be avoided by using the silicates [4,171,172].

**Figure 48:**
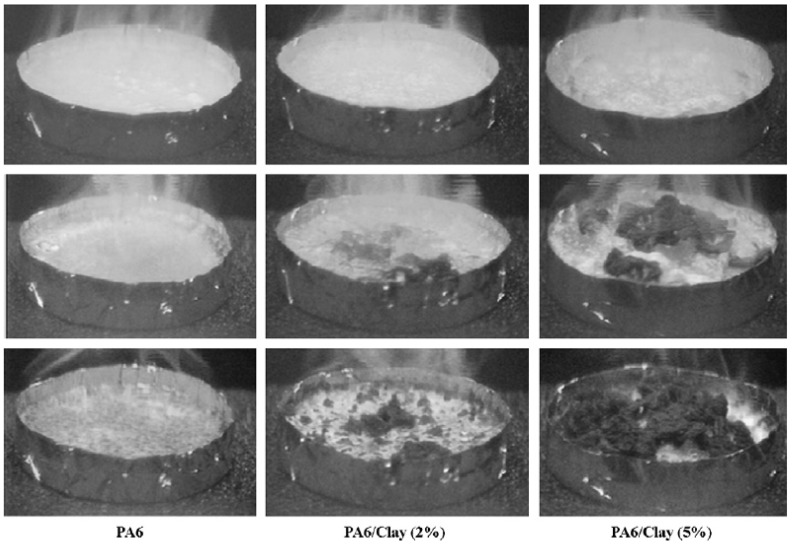
Flame behavior of the pure nylon, nylon nanocomposites with 2 and 5% of clay. The images have been taken at 100, 200 and 400s of the process. Reproduced from reference [173] with permission from Elsevier.

Kashiwagi *et al.* reported the flame retardancy properties of polyamide nanocomposites containing 2 and 5% of clay [173]. The authors observed that the specific heat of combustion (H_c_) (which is obtained from the ratio of HRR to MLR) remains unchanged for the three samples. From this observation, it was suggested that the observed reduction in HRR (and MLR) is due to chemical and physical processes in the condensed phase, rather than in the gas phase. The authors also subjected the pure polymer and nanocomposite samples to heat flux in the cone calorimeter, but in nitrogen. It was observed the overall differences among the three samples were very similar between the burning case and the gasification case, even though there were quantitative differences in MLR between the two cases. As the gasification behavior is dependant only on processes in the condensed phase, it was suggested by the authors that the observed improvement in the flammability resistance of nanocomposites is due to chemical and physical processes in the condensed phase. As visible in Figure 48, the nanocomposites appeared to be more viscous than the neat PA6 sample during gasification. Dark floccules appeared on the surfaces of nanocomposite materials which grew with time.

### 5.4. Crystallinity

It is also well known that the commonly used reinforcing fillers used for polypropylene like talc and calcium carbonate can also act as strong nucleating agents, thus affecting the degree of crystallinity, rate of crystallization, crystal size and lamellae orientation [174,175]. Such changes in crystallization behavior have a strong impact on the properties of the composite. Clay particles can also act as nucleating agents depending on the processing conditions used, degree of dispersion and surface coverage [176,177]. Maiti *et al.* reported the decrease of spherulite size with increase in clay content [178]. Presence of tactoids owing to poor dispersion of the filler was also reported to cause a decrease in the spherulite size [176]. Kodgire *et al.* reported that PP showed advanced crystallization and fibrous morphology rather than usual spherulite behavior in the presence of clay [179]. Similarly, decrease in crystallinity and increase in nucleus density was also observed in other studies owing to the nucleation effect of the clay platelets [180].

Microscopic investigations of the polymer nanocomposites have also been further developed to gain better insights into the morphology of the composite particles. Apart from transmission electron microscopy commonly used, the use of atomic force microscopy in studying the interactions of the organic and inorganic phases has also been found to be beneficial. 

## 6. Summary

Nanocomposites have attracted a tremendous research effort in the last two decades owing to achievement of the significant improvement in the composite properties at a very low filler fraction. Toyota researchers in their studies in early nineties used the *in-situ* intercalative polymerization approach for the synthesis of polyamide nanocomposites. Since then, a number of synthesis methods have been developed and nanocomposites with a large number of polymer matrices have been reported. Giannelis and co-workers reported melt intercalation for the polymer nanocomposite synthesis where there is no requirement of the solvent for the composite generation. Other synthesis methods include intercalation of polymer or pre-polymer from solution and template synthesis. Based on the composite morphology, the nanocomposites are generally classified as intercalated or exfoliated, but such ideal classification is arbitrary in many cases owing to the presence of mixed intercalated and exfoliated morphology in the composites. The exfoliated platelets are the most significant contributors to the enhancement in the composite properties, as the intercalated platelets do not increase the aspect ratio of the platelets. The interactions of the silicate surface, surface modification as well as polymer with each other define if the filler would be exfoliated in the polymer matrix or not. Apart from that, thermally stable surface modification and filler surface free off any excess surface modification are also required for optimal interactions. In the case of polar polymers, the polymer chains can interact with the polar silicate surface thus leading to extensive exfoliation of the filler in the polymer. These interactions also overcome the unattractive interactions between the polar polymer chains and the non-polar surface modification chains. However, in the case of non-polar polymers, there are no interactions present between the phases of the composites. Partial polarization of the matrix is generally achieved by the addition of low molecular weight amphiphilic compatibilizers. Other route is to increase the basal plane spacing of the filler as much as possible by using advanced surface modification methods. This leads to the reduction of the electrostatic forces of attraction holding the platelets together thus allowing the delamination of the platelets kinetically by shear in the compounder. Modulus of the composites is generally observed to increase owing to the incorporation of clay, however, the contradictory results have been presented for the tensile and impact strength and these properties are more dependant on the resulting composite morphology. The barrier properties of the nanocomposites are also significantly improved, but it is required to consider the compatibility of the organic and inorganic phases at interface. The thermal stability as well as flame retardancy of the polymer materials also increases on the incorporation of the small amount of filler. The use of silicate materials as flame retardants in the polymer composites is more environmentally friendly approach than the conventionally used flame retardants. 
